# Ultrafast Electron Transfer Coupled with a Proton Relay in an Anisotropic Dual S‐Scheme Heterojunction for Overcoming Kinetics Mismatch in H_2_O_2_ Photosynthesis

**DOI:** 10.1002/advs.202522285

**Published:** 2025-12-23

**Authors:** Bing Wang, XiangBo Feng, Yao Liu, XinYi Wang, EnZhou Liu, YuZhen Zhao, ZongCheng Miao, Zhuo Li

**Affiliations:** ^1^ Shaanxi Key Laboratory of Liquid Crystal Polymer Intelligent Display Technological Institute of Materials & Energy Science (TIMES) School of Computer Science Xijing University Xi'an P. R. China; ^2^ School of Chemical Engineering Northwest University Xi'an P. R. China; ^3^ School of Artificial Intelligence, Optics and Electronics (iOPEN) Northwestern Polytechnical University Xi'an China

**Keywords:** anisotropic dual S‐scheme heterojunction, electron–proton kinetics mismatch, ultrafast electron transfer, proton relay, photosynthetic H_2_O_2_ production

## Abstract

The kinetic mismatch between electron transfer and proton diffusion fundamentally limits the efficiency of photocatalytic H_2_O_2_ production. To address this, an anisotropic dual S‐scheme heterojunction (C_3_N_4_/SubPc‐1/C_3_N_5_) is constructed to achieve spatiotemporal synergy between charge and proton transport. This multidimensional design establishes a tridirectional (lateral, vertical, and internal) charge transfer network, enabling ultrafast electron migration. Simultaneously, the ─CONH─ bridge acts as a dual channel for concurrent electron and proton transfer. Coupled with a Yeager‐type oxygen adsorption configuration that preferentially activates a dual‐pathway 2e^−^ oxygen reduction reaction (ORR), the optimized catalyst achieves an exceptional H_2_O_2_ production rate of 2048.7 µmol·g^−^^1^·h^−1^ and an apparent quantum yield of 16.28% at 400 nm. A combination of synchrotron radiation X‐ray photoelectron spectroscopy (SI‐XPS), femtosecond transient absorption spectroscopy (fs‐TAS), and multiscale theoretical calculations —including density functional theory (DFT), time‐dependent density functional theory (TDDFT), and molecular dynamics (MD) simulations— collectively reveals that the <1 ps anisotropic dual S‐scheme electron transfer mechanism works synergistically with the proton relay function to efficiently drive charge separation and reactant activation. This study provides a universal interfacial engineering paradigm for managing complex proton‐coupled electron transfer (PCET) processes in artificial photosynthesis.

## Graphical abstract

An anisotropic dual S‐scheme heterojunction (C_3_N_4_/SubPc‐1/C_3_N_5_) is constructed to overcome the electron–proton kinetics mismatch in photocatalytic H_2_O_2_ production. The imide bridge serves as a dual channel for ultrafast electron transfer and proton relay, synergistically enabling efficient dual‐pathway oxygen reduction and achieving a high H_2_O_2_ production rate of 2048.7 µmol g^−1^ h^−1^.

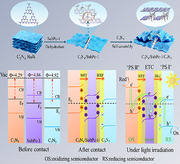

## 1 Introduction

Hydrogen peroxide (H_2_O_2_) is a sustainable green oxidant and clean energy carrier with broad application prospects in environmental remediation, chemical synthesis, and biomedicine [1, 2, 3, 4, 5]. However, the current industrial anthraquinone process is energy‐intensive, relies on toxic organic solvents, and involves complex operations, making the development of efficient and sustainable solar‐driven photocatalytic alternatives highly imperative [6, 7, 8, 9]. Among various candidate materials [10], graphitic carbon nitride (g‐C_3_N_4_) and its derivatives are regarded as an ideal platform for photocatalytic H_2_O_2_ production due to their tunable electronic band structures and high stability [11, 12, 13, 14, 15]. Nevertheless, g‐C_3_N_4_ suffers from inherent limitations such as high exciton binding energy and strong coulomb interactions, which hinder exciton dissociation and lead to severe charge recombination [16, 17]. Moreover, the interlayer energy barrier impedes the cross‐plane migration of photogenerated charges, which preferentially migrate along the in‐plane direction within the π‐conjugated layered structure [18].

Although various modified g‐C_3_N_4_‐based systems have achieved photocatalytic H_2_O_2_ synthesis in recent years, their overall efficiency remains limited by the intrinsic kinetics of the oxygen reduction reaction (ORR) [19, 20, 21]. Mechanistically, the reduction of O_2_ to H_2_O_2_ involves the sequential transfer of two electrons and two protons [22, 23]. However, the ultrafast recombination of photogenerated carriers in g‐C_3_N_4_—occurring on picosecond to nanosecond timescales—contrasts sharply with the relatively slow proton diffusion in aqueous media (approximately 2.39 × 10^−^⁹ m^2^ s^−1^ at 300 K) [24, 25, 26], resulting in a significant kinetic mismatch between electron and proton supply [6]. Thus, resolving this electron–proton kinetic mismatch is crucial for enhancing the photocatalytic H_2_O_2_ production performance of g‐C_3_N_4_. This kinetic barrier not only limits the reaction rate but also compromises the selectivity of the two‐electron (2e^−^) ORR pathway.

**SCHEME 1:**
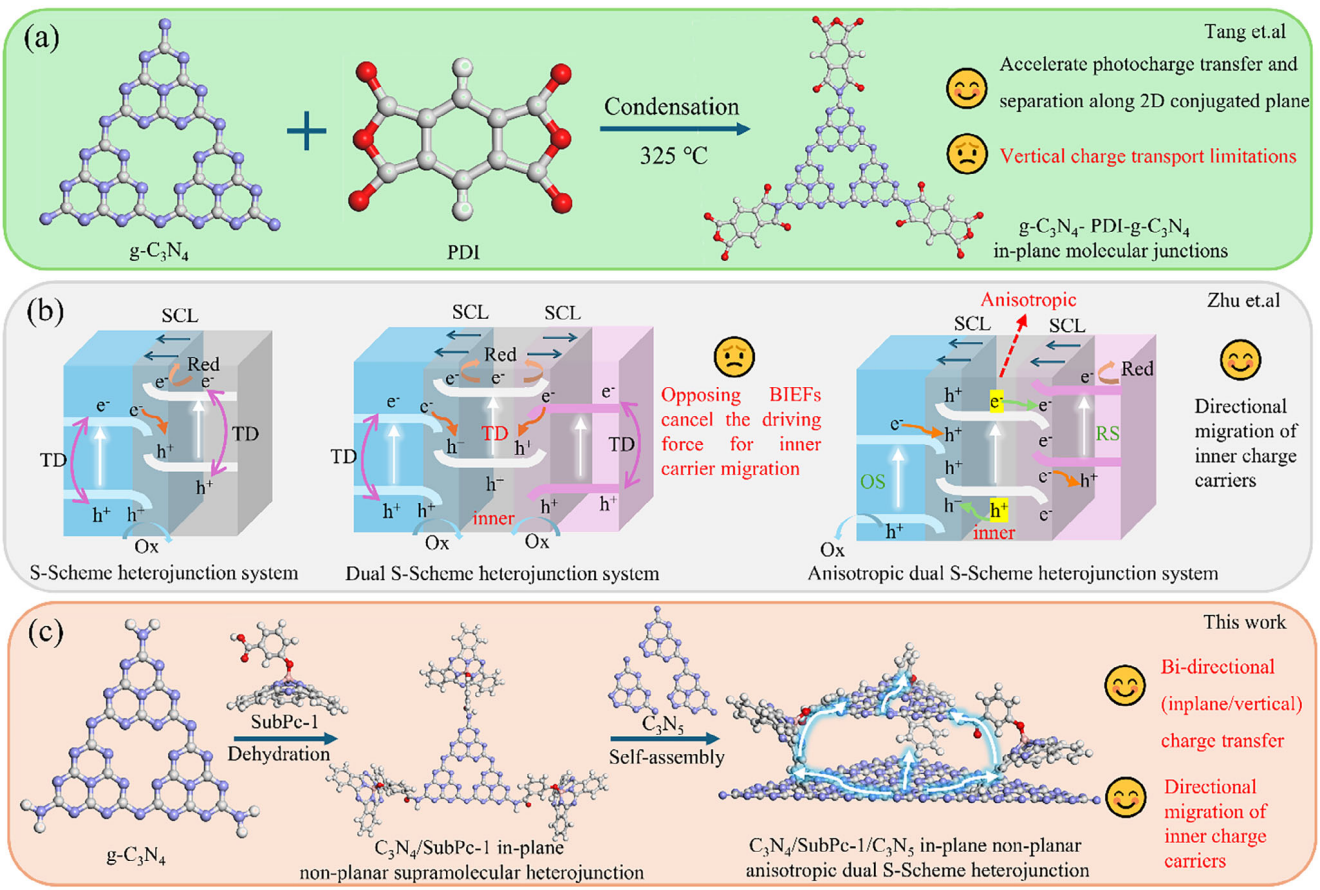
(a) Synthetic route for g‐C_3_N_4_─PDI─g‐C_3_N_4_ in‐plane molecular junctions. (b) Anisotropic dual S‐scheme heterojunction system. (c) Synthetic route for C_3_N_4_/SubPc‐1/C_3_N_5_ in‐plane nonplanar anisotropic dual S‐scheme heterojunction.

To address these challenges, heterojunction engineering has emerged as a promising strategy for improving photocatalytic performance [27]. In‐plane junctions can enhance in‐plane charge transfer and separation within the 2D conjugated planes of g‐C_3_N_4_ [18, 28]. For instance, Tang et al. constructed a g‐C_3_N_4_/PDI‐g‐C_3_N_4_ in‐plane homojunction [29], which facilitated in‐plane carrier separation and transport; however, the layered stacking structure still hindered the migration of charges toward interfacial regions and catalyst surfaces (Scheme 1a). On the other hand, S‐scheme heterojunctions have attracted extensive attention due to their strong built‐in electric field (BIEF), directed charge migration, and retained strong redox capabilities [30, 31, 32]. Yet, conventional S‐scheme systems are constrained by the limited distribution of the interfacial space charge layer (SCL) [33, 34, 35]. In regions beyond the SCL, carriers migrate via thermodynamic diffusion, leading to substantial recombination (Scheme 1b). Moreover, in traditional dual S‐scheme heterojunctions, the presence of two oppositely oriented BIEFs counteracts the driving force for directional carrier migration (Scheme 1b) [36, 37]. Recently, Zhu and co‐workers reported an anisotropic dual S‐scheme heterojunction composed of Bi_2_O_3_─BiOBr─AgI. By leveraging the anisotropic carrier migration within BiOBr, an electron transport chain (ETC)‐like pathway was constructed, effectively suppressing charge recombination (Scheme 1b) [38]. Despite these advances, a critical scientific challenge remains: how to simultaneously achieve rapid lateral migration while establishing efficient vertical charge channels and introducing intrinsic active sites.

Inspired by the natural photosynthetic ETC, this study proposes a multidimensional biomimetic design strategy (Scheme 1c). First, an in‐plane S‐scheme heterojunction was constructed via imide bond (─CONH─) formation between terminal amino groups of g‐C_3_N_4_ and axially substituted carboxyl‐functionalized subphthalocyanine H_12_SubPcB‐OPhCOOH (SubPc‐1), enabling precise regulation of lateral charge migration. Subsequently, C_3_N_5_ was introduced to couple with the g‐C_3_N_4_/SubPc‐1 system, forming an anisotropic dual S‐scheme heterojunction (g‐C_3_N_4_/SubPc‐1/C_3_N_5_) for synergistic regulation of both lateral and vertical charge migration. Notably, the imide bond not only serves as an electron bridge but also facilitates proton transfer via its N─H sites. Concurrently, the Yeager‐type O_2_ adsorption configuration enables simultaneous direct and indirect 2e^−^ ORR pathways. This multidimensional coupling strategy integrates six key advantages: the construction of an anisotropic dual S‐scheme heterojunction and the establishment of an in‐plane nonplanar junction significantly optimize charge separation and migration pathways; proton transfer is enhanced via the ─CONH─ bond, while both direct and indirect 2e^−^ ORR are enabled through Yeager‐type O_2_ adsorption; together, these innovations accomplish highly efficient photosynthetic H_2_O_2_ production and facilitate synergistic degradation of organic pollutants such as rhodamine B (RhB). Through precise molecular‐interface engineering, this work realizes, for the first time, the synergistic coupling of multidimensional charge regulation and reaction pathways within a single system, overcoming the electron–proton kinetic mismatch bottleneck in ORR. It provides a novel strategy for efficient photosynthetic H_2_O_2_ production and organic pollutant degradation, while also broadening the application prospects of g‐C_3_N_4_‐based materials in sustainable energy and environmental remediation.

## 2 Results and Discussion

### 2.1 Synthesis and Characterization

**FIGURE 1:**
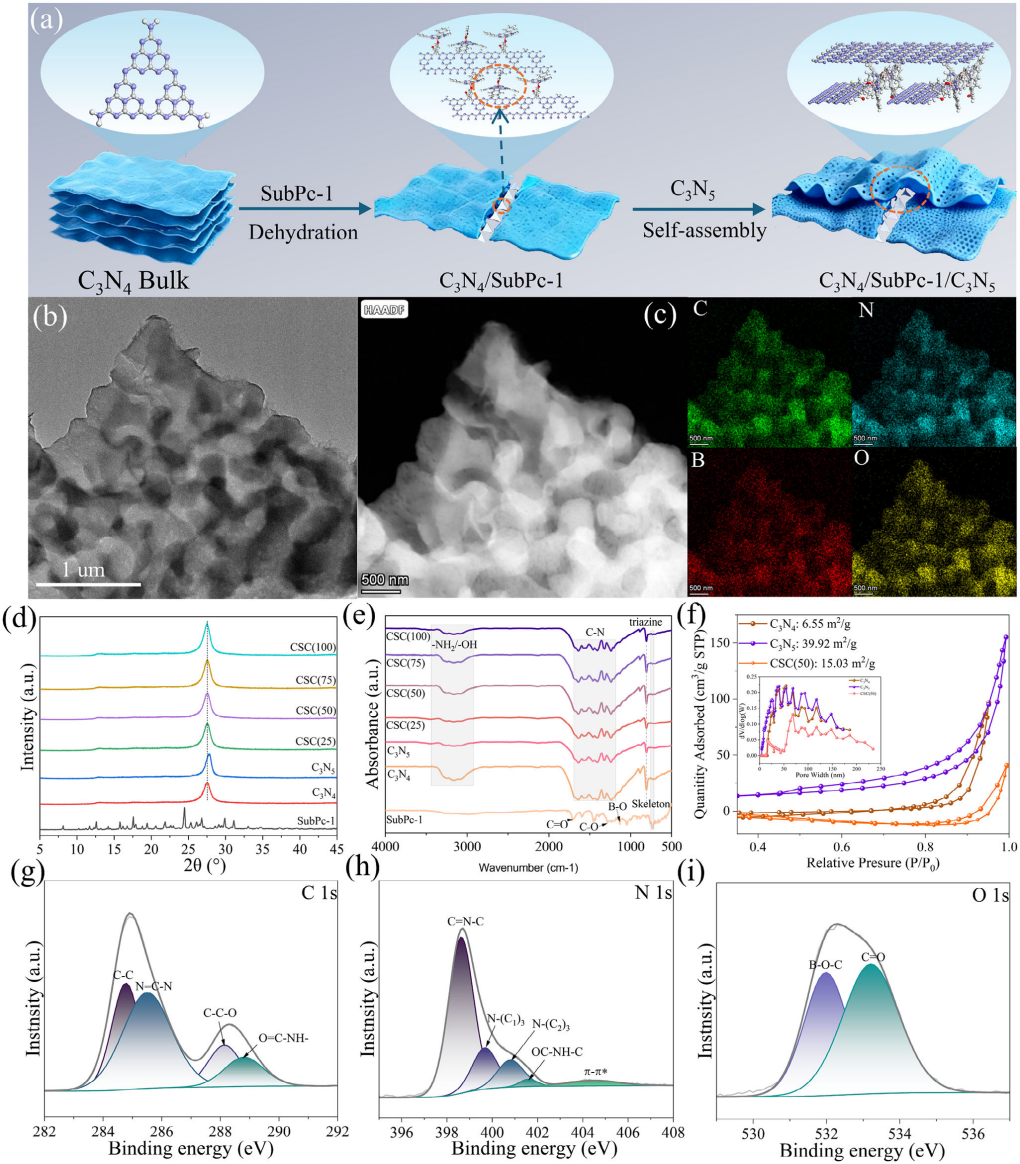
(a) Schematic illustration of the preparation of C_3_N_4_/SubPc‐1/C_3_N_5_, (b) TEM image of CSC(50), (c) HAADF‐STEM image of CSC(50) and the corresponding elemental mapping, (d–f) XRD patterns, FT‐IR spectra, and BET isotherms of the as‐prepared materials, high‐resolution XPS spectra of (g) C 1s, (h) N 1s, and (i) O 1s.

The synthesis process of the C_3_N_4_/SubPc‐1/C_3_N_5_ (CSC) heterostructure is illustrated in Figure 1a. Initially, SubPc‐1 is anchored onto C_3_N_4_ via an amidation reaction between the axial carboxyl group of SubPc‐1 and the terminal amino groups present on C_3_N_4_, resulting in the formation of a nonplanar in‐plane junction. At the linkage sites, the SubPc‐1 molecules further undergo self‐assembly through π–π stacking interactions, leading to the formation of supramolecular structures [39, 40]. Subsequent introduction of C_3_N_5_ induces wrinkling on the catalyst surface due to the nonplanar geometry of the C_3_N_4_/SubPc‐1 junction, resulting in distorted nanosheets with locally reduced interlayer spacing. This structural modification facilitates enhanced vertical charge transport, mitigating the limitations inherent in bulk C_3_N_5_. SEM images reveal that pristine C_3_N_4_ exhibits a highly aggregated nanosheet morphology (Figure S2a, Supporting Information), while SubPc‐1 consists of micrometer‐sized bulk particles (Figure S2b, Supporting Information), and C_3_N_5_ appears as agglomerated blocks (Figure S2c, Supporting Information). In contrast, TEM imaging of the CSC composite (Figure 1b) shows that twisted C_3_N_5_ nanosheets are uniformly supported on thin C_3_N_4_ layers. Furthermore, HAADF‐STEM analysis (Figure 1c) reveals a porous surface architecture, and elemental mapping confirms the homogeneous distribution of SubPc‐1 on the nanosheet surfaces. The molecular architecture of SubPc‐1 was unambiguously determined by single‐crystal X‐ray diffraction analysis. Its solid‐state conformation and crystalline packing arrangement are depicted in Figures S3 and S4 (Supporting Information). Essential crystallographic refinement data for the acquired single crystal are comprehensively itemized in Tables S1 and S2 (Supporting Information), respectively. The SubPc‐1 molecule adopts a nearly cone‐shaped geometry centered around a boron atom [41]. The central boron atom is coordinated with three isoindoline nitrogen atoms and one axial oxygen atom (from the 3‐carboxymethylphenoxy group), forming a stable “3N + OPhCOOH” coordination environment [42]. The crystal belongs to the monoclinic system, with two molecules per unit cell and a crystal density of 1392 kg m^−^^3^. The unit cell parameters are: *a* = 9.047(3) Å, *b* = 12.868(7) Å, *c* = 12.993(4) Å; *α* = 106.868(8)°, *β* = 108.918(6)°, *γ* = 99.407(9)°; the cone depth is 2.6524(99) Å. Structure refinement unambiguously confirms that the axial 3‐carboxymethylphenoxy group replaces the bromine atom via its hydroxyl group, forming a B─O single bond with a length of 1.446(8) Å. The B─N bond lengths range from 1.477(9) to 1.530(8) Å. The central boron atom deviates from the plane defined by the three isoindoline nitrogen atoms by a vertical distance of 0.6268(99) Å. The bond angles O(1)─B(1)─N(3), O(1)─B(1)─N(1), and O(1)─B(1)─N(5) are 117.6(6)°, 118.7(6)°, and 107.3(5)°, respectively, indicating that the axial substituent is slightly tilted toward the N(5) atom. In the solid‐state packing, SubPc‐1 molecules form intramolecular dimers via a “convex‐to‐convex, head‐to‐head” arrangement [43, 44]. Within such a dimer, the isoindoline six‐membered ring substructure (C10─C11─C12─C13─C14─C15) of one SubPc‐1 molecule exhibits intramolecular π–π stacking interactions with both the five‐membered ring (N3─C9─C10─C15─C16) and the six‐membered ring (C10─C11─C12─C13─C14─C15) substructures of an adjacent molecule, with centroid distances of 3.831 and 3.811 Å, respectively. The distance between the boron centers is 9.3568(94) Å. All molecules extend infinitely in space through identical interactions, forming a continuous π‐stacking array. The strategic construction of supramolecular assemblies with extended π‐arrays serves to markedly prolong the excited‐state persistence of photosensitizers while concurrently improving their charge transport characteristics. This synergistic enhancement directly facilitates more efficient interfacial charge transfer to catalytic sites, culminating in a substantial boost to overall photocatalytic activity [40].

Structural analysis via X‐ray diffraction (Figure 1d) confirmed the crystalline phases of the pristine components and their composites. The XRD profile of pure C_3_N_4_ is readily identifiable by two characteristic signals: the (100) peak at 13.0° (*d* = 0.68 nm), attributed to the in‐plane structural periodicity of s‐heptazine rings [45], and the intense (002) peak at 27.1° (*d* = 0.32 nm), which originates from the interlayer stacking of the conjugated nanosheets [46]. In contrast, pure C_3_N_5_ shows a distinct diffraction peak at 2*θ* = 27.6°, which corresponds to a smaller interplanar spacing compared to that of C_3_N_4_ [47]. This difference may be due to the presence of azo (─N═N─) bridging groups, which enhance the π‐conjugation within C_3_N_5_ layers and strengthen the interlayer interactions between adjacent π‐conjugated systems. Additionally, the decreased intensity of the (100) peak in C_3_N_5_ suggests a more disordered in‐plane arrangement of the s‐heptazine units [48]. No identifiable crystalline phase from SubPc‐1 was detected in the composite materials, a finding attributable to its exceptionally low concentration (<4 wt%) and molecular‐level dispersion within the host matrix [49]. The high crystallinity and phase purity of the CSC composites are corroborated by the presence of sharp, intense diffraction patterns devoid of any extraneous peaks.

The chemical structures of the synthesized materials were further probed by Fourier‐transform infrared (FTIR) spectroscopy, as displayed in Figure 1e. The spectrum of pristine SubPc‐1 is featured by a characteristic peak near 1600 cm^−1^, assigned to C═C bond stretching, and another at approximately 1500 cm^−1^, attributed to C═N stretching vibrations [42]. Additional diagnostic bands were identified at 1133 cm^−^1 (B─O vibration), 1285 cm^−1^ (C─O stretch), and 1710 cm^−1^ (C═O stretch) [42]. For C_3_N_4_, a broad infrared band around 3145 cm^−1^ is attributable to stretching vibrations of residual ─NH_2_/─OH groups [46]. Additional spectral features confirmed the triazine structure: an out‐of‐plane bending vibration was observed at 806 cm^−1^, while the complex set of bands in the 1200–1600 cm^−1^ range, along with a specific bending mode at 885 cm^−1^, are attributable to the stretching and deformation vibrations of the triazine rings, respectively [46]. C_3_N_5_ shows functional groups similar to those of C_3_N_4_; notably, the absence of a peak at 1075 cm^−1^ in C_3_N_5_ confirms structural differences in their frameworks. In all CSC composites, in addition to features similar to C_3_N_4_, a band at 730 cm^−1^ corresponding to the macrocyclic skeletal vibration of SubPc‐1 is detectable. Moreover, as the content of SubPc‐1 increases, the intensity of the ─NH_2_/─OH band decreases in the composites, which is consistent with the consumption of amine groups due to amide bond formation. BET measurements indicate that the CSC(50) composite possesses a larger specific surface area compared to pure C_3_N_4_, along with a reduction in average pore size (Figure 1f). This can be attributed to the partial incorporation of SubPc‐1 molecules into the pore channels, which increases surface roughness and pore complexity while simultaneously constraining the pore dimensions.

To probe the surface composition and bonding states, X‐ray photoelectron spectroscopy (XPS) analysis was performed. The survey scan verified the presence of carbon, nitrogen, oxygen, and boron in the CSC(50) composite (Figure S5, Supporting Information). Deconvolution of the high‐resolution C 1s spectrum (Figure 1g) yielded four distinct components with binding energies of 284.8, 285.5, 288.1, and 288.8 eV. These are assigned to C─C bonds, N═C─N within the heptazine framework, C─C─O from SubPc‐1 [42], and O─C─NH─ from the amide bond [50], respectively. The N 1s spectrum exhibits five fitted peaks (Figure 1h): π–π* excitations at 404.5 eV, ─OC─NH─C from the amide bond at 401.6 eV, N─(C)_3_ bridging s‐heptazine units at 400.8 eV, N─(C)_3_ connecting tri‑s‑triazine units at 399.7 eV, and C─N═C at 398.6 eV [46]. In the O 1s spectrum, the peaks located at 532.0 and 533.2 eV are attributed to B─O─C from SubPc‐1 and C═O from the amide functionality [42, 46], respectively (Figure 1i). Furthermore, the B 1s spectrum shows two characteristic peaks at 182.8 and 191.4 eV, assigned to N─B and B─O bonds in SubPc‐1 (Figure S6, Supporting Information) [42].

**FIGURE 2:**
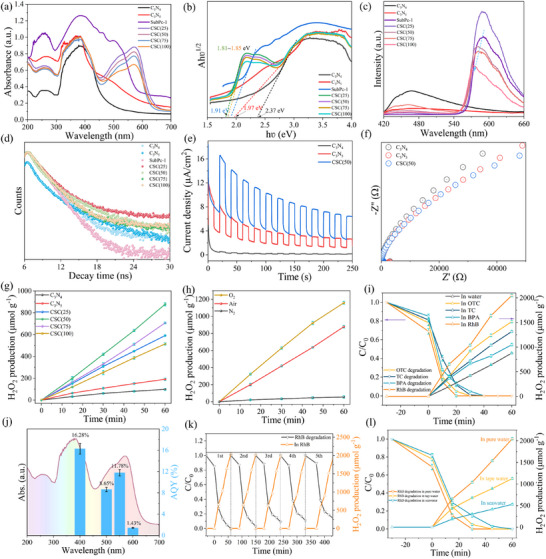
(a) UV–Vis DRS spectra, (b) Tauc plots, (c) PL spectra, (d) Time‐resolved PL decay curves, (e) transient photoresponse responses, (f) EIS Nyquist plots of the as‐prepared materials. (g) The photocatalytic H_2_O_2_ production performance of the as‐prepared catalysts, (h) photocatalytic H_2_O_2_ production in different gas atmosphere over CSC(50). (i) Photocatalytic production of hydrogen peroxide synergistically degrading various pollutants. (j) Wavelength‐dependent apparent quantum yield (AQY) for photocatalytic H_2_O_2_ production in a 20 mg L^−1^ RhB aqueous solution (50 mL RhB, 50 mg catalyst, 1 g L^−1^ catalyst loading, 25 °C). (k) Five‐cycle experiment on synergistic photocatalytic H_2_O_2_ production and RhB degradation with CSC(50). (l) CSC(50)‐mediated photocatalytic H_2_O_2_ production and RhB degradation in pure water, tap water, and seawater.

As shown in the ultraviolet–visible diffuse reflectance spectroscopy (UV–vis DRS) spectra in Figure 2a, pristine C_3_N_4_ exhibits strong absorption below 450 nm, which can be attributed to the π–π* electronic transitions within its s‐triazine or s‐heptazine structural motifs [51]. In contrast, pure C_3_N_5_ displays a distinct redshift in absorption toward longer wavelengths, likely resulting from an extended π‐conjugation network facilitated by orbital overlap between the N 2p states of the azo‐bridging groups and the π‐system of the s‐heptazine units [52]. Pristine SubPc‐1 shows intense absorption below 560 nm, corresponding to its characteristic B‐band and Q‐band electronic transitions [42]. All CSC composites demonstrate significantly enhanced absorption across the visible region (480–620 nm). This enhancement can be ascribed to the synergistic effects among the components, including improved light‐harvesting due to the incorporation of SubPc‐1, facilitated charge transfer across the heterojunction interfaces—promoted by the amide bonding between C_3_N_4_ and SubPc‐1—and electronic coupling within the integrated supramolecular π‐system composed of C_3_N_4_, SubPc‐1, and C_3_N_5_. Tauc plot analysis (Figure 2b) quantified the optical bandgaps of C_3_N_4_ (2.37 eV), C_3_N_5_ (1.97 eV), and SubPc‐1 (1.91 eV). A noticeable reduction in bandgap was observed across all composite materials, ranging from 1.81 to 1.85 eV.

Steady‐state photoluminescence (PL) measurements were carried out to evaluate charge recombination. A prominent fluorescence quenching effect is observed for all CSC ternary composites compared to the pristine materials (Figure 2c). This attenuation of the PL signal points to the prevalence of nonradiative decay pathways, indicative of more efficient photogenerated charge separation [42]. Notably, the emission peaks of all CSC composites are blueshifted relative to those of the individual components, which may originate from interfacial electronic interactions mediated by the cascaded chemical bonding—specifically the amide linkage—among the three constituents [42]. To elucidate the origin of the enhanced performance of the CSC composites, time‐resolved photoluminescence (TRPL) measurements were further carried out (Figure 2d). The corresponding fluorescence decay profiles were fitted with a biexponential decay model, and the detailed lifetime parameters are summarized in Table S3 (Supporting Information). Notably, the CSC composites exhibit prolonged average lifetimes (*τ*_av_) compared to the pristine components. While CSC(25) shows the longest *τ*_av_ (28.45 ns), CSC(50) achieves the optimal balance with a significantly extended *τ*_av_ of 25.08 ns compared to C_3_N_4_ (15.2 ns), C_3_N_5_ (16.4 ns), and SubPc‐1 (14.24 ns). This suggests that although higher SubPc‐1 content further suppresses charge recombination, optimal photocatalytic performance requires an appropriate balance between carrier lifetime and accessible active sites. The extended carrier lifetime in CSC(50), attributed to the facilitated interfacial charge transfer across the well‐built heterojunctions, is consistent with its enhanced photocatalytic performance, further corroborating the effectiveness of the proposed material design [42]. The transient photocurrent responses of the as‐prepared materials are shown in Figure 2e. CSC(50) exhibits the highest photocurrent density, indicating efficient interfacial charge separation and transfer [53]. Furthermore, electrochemical impedance spectroscopy (EIS) reveals that CSC(50) possesses a smaller arc radius than pristine C_3_N_4_, suggesting minimized interfacial charge transfer resistance and faster kinetics at the electrode–electrolyte interface (Figure 2f) [54]. This coherent set of data highlights the multifold improvements in charge dynamics: the photocurrent, sensitive to overall charge separation and utilization, shows a dramatic increase due to superior bulk separation, while the EIS confirms the concomitant optimization of interfacial charge transfer kinetics.

### 2.2 Photocatalytic Performance Analyses

A systematic investigation was conducted to evaluate the photocatalytic performance of C_3_N_4_, C_3_N_5_, and CSC composites for H_2_O_2_ production and synergistic pollutant degradation. Owing to their low charge separation efficiency, the pristine C_3_N_4_ and C_3_N_5_ achieved H_2_O_2_ production rates of only 98.2 and 190.6 µmol·g^−1^·h^−1^, respectively. In contrast, all composite materials exhibited enhanced performance, with CSC(50) reaching a high H_2_O_2_ generation rate of 878.4 µmol·g^−1^·h^−1^, which is 8.95 and 4.61 times that of C_3_N_5_ and C_3_N_4_, respectively (Figure 2g). This marked enhancement in performance stems from the enhanced charge carrier separation enabled by the heterojunction interfaces in the CSC composite. Furthermore, the effect of the reaction atmosphere on H_2_O_2_ synthesis was investigated (Figure 2h). When operated under a pure O_2_ environment, the H_2_O_2_ production rate for CSC(50) increased to 1158.4 µmol·g^−1^·h^−1^, representing a 1.32‐fold enhancement over the performance in air. Conversely, switching to a N_2_ atmosphere resulted in negligible activity, unequivocally verifying that molecular oxygen is essential and that H_2_O_2_ generation occurs predominantly via the ORR route. To gain deeper mechanistic insight into the superior performance, particularly concerning the role of the ─CONH─ bridge in facilitating proton transfer—a critical step in the ORR pathway—we performed comparative H/D kinetic isotope effect (KIE) measurements. A control sample, designated as PM‐CSC(50), was prepared via mechanical grinding to maintain an identical chemical composition to the covalent CSC(50) heterojunction while precluding the formation of amide bonds. The results provided compelling evidence: the covalent CSC(50) exhibited a low KIE value of 1.27, whereas the physical mixture PM‐CSC(50) showed a significantly higher KIE of 2.02 (Figure S7, Supporting Information). This stark contrast unambiguously demonstrates the critical role of the covalent amide linkage beyond simple compositional effects. The high KIE value for the physical mixture identifies slow, diffusion‐limited proton transfer as the dominant kinetic bottleneck in the absence of a designed proton channel. In contrast, the drastically reduced KIE value for CSC(50), which is characteristic of a Grotthuss‐type proton hopping mechanism, confirms that the ─CONH─ covalent bridge serves as an efficient biomimetic proton relay [55]. This preorganized channel facilitates rapid proton shuttling, effectively alleviating the kinetic bottleneck and thereby substantially enhancing the overall reaction efficiency for H_2_O_2_ production.

To assess the potential for practical implementation, we performed concurrent H_2_O_2_ production and pollutant decomposition tests in simulated wastewater contaminated with various organics, including oxytetracycline (OTC), tetracycline (TC), bisphenol A (BPA), or RhB (Figure 2i). Notably, compared to performance in pure water, the H_2_O_2_ evolution rate of CSC(50) increased significantly in the different pollutant systems, reaching 1517 (OTC), 1315 (TC), 1048.4 (BPA), and 2048.7 (RhB) µmol·g^−1^·h^−1^, respectively. All pollutants were completely degraded within 40 min. To further elucidate the role of organic additives and exclude potential dye‐sensitization effects, control experiments were conducted using nonabsorbing sacrificial agents. The H_2_O_2_ production rates followed the order: Methanol (1320.5 µmol·g^−1^·h^−1^) > Ethanol (1150.8 µmol·g^−1^·h^−1^) > Pure Water (878.4 µmol·g^−1^·h^−1^) > isopropanol (IPA) (680.3 µmol·g^−1^·h^−1^) > TEOA (420.6 µmol·g^−1^·h^−1^) (Figure S8, Supporting Information). The superior performance of methanol and ethanol confirms their effectiveness as hole scavengers, while the reduced activity with IPA and TEOA—even lower than in pure water—suggests that strong competitive adsorption on active sites or undesirable side reactions may partially inhibit the oxygen reduction process despite their hole‐scavenging capability. The exceptional performance in the RhB system, significantly surpassing all other conditions, reveals its unique dual functionality. This synergistic enhancement can be attributed to the dual role of organic pollutants: acting as hole scavengers that consume photogenerated holes to suppress charge recombination, while also serving as proton donors, thereby facilitating H_2_O_2_ generation. The superior synergy observed with RhB is attributed to its unique ability to optimize the interfacial charge transfer dynamics, as inferred from the contact angle data (Figure S9, Supporting Information). The ultralow contact angle (14.37°) indicates strong, preferential adsorption of cationic RhB onto the catalyst surface, creating a highly hydrophilic interface [6]. This configuration facilitates the rapid scavenging of photogenerated holes (h⁺) by the preadsorbed RhB molecules. The efficient consumption of h⁺, the primary recombination centers, in turn promotes the separation of charge carriers and directs the photogenerated electrons (e^−^) toward the more efficient 2e^−^ ORR for H_2_O_2_ production. Thus, the RhB degradation cycle directly enhances the H_2_O_2_ synthesis pathway, creating a positive feedback loop. In contrast, the larger contact angles for antibiotics (e.g., 56.64° for OTC) suggest weaker adsorption and a less favorable interface, leading to inefficient hole consumption and consequently inferior charge separation and H_2_O_2_ yield. Furthermore, as a typical dye molecule, RhB can also act as an electron donor in the photocatalytic process, further promoting the ORR pathway and increasing the H_2_O_2_ yield.

To further assess the efficiency of light utilization, the apparent quantum yield (AQY) for H_2_O_2_ production was monitored at specific wavelengths. The CSC(50) composite demonstrated AQYs of 16.28%, 8.65%, 11.78%, and 1.43% when irradiated at 400, 500, 550, and 600 nm, respectively (Figure 2j). The observed wavelength‐dependent response correlates directly with the material's absorption spectrum, validating its capability to efficiently convert photon energy into chemical potential. The photocatalytic performance of the CSC(50) catalyst was systematically evaluated and compared with both our previously reported single S‐scheme C_3_N_4_/C_3_N_5_ heterojunction and other state‐of‐the‐art photocatalysts for H_2_O_2_ production (Table 1). Particularly noteworthy is the significant enhancement achieved by the anisotropic dual S‐scheme design. Compared to the single S‐scheme C_3_N_4_/C_3_N_5_ heterojunction (446 µmol·g^−1^·h^−1^ in TC solution), the CSC(50) catalyst demonstrates approximately twofold higher activity in pure water (878.4 µmol·g^−1^·h^−1^) and 4.6‐fold enhancement in the RhB system (2048.7 µmol·g^−1^·h^−1^). This remarkable improvement highlights the superior charge separation efficiency and proton transfer capability of the dual S‐scheme architecture. Furthermore, the CSC(50) catalyst exhibits competitive performance among advanced photocatalytic systems, particularly in terms of apparent quantum yield. The high AQY of 16.28% at 400 nm and the substantial production rate in pure water underscore the effectiveness of the anisotropic dual S‐scheme heterojunction design coupled with the proton relay functionality.

**TABLE 1:** Performance comparison of CSC(50) with state‐of‐the‐art photocatalysts for H_2_O_2_ production.

Photocatalyst	Light Source (300 W Xe lamp)	React Solution	H_2_O_2_ Yield (µmol·g^−1^·h^−1^)	AQY (%)	References
CSC(50)	*λ* ≥ 400 nm 350 mW cm^−2^	RhB (20 mg L^−1^)	2048.7	16.28 (400 nm)	this work
BWO/TCN‐Gd	*λ* ≥ 420 nm 100 mW cm^−2^	Pure water	749.2	11.3 (420 nm)	[56]
CoOx‐BCN‐FeOOH	*λ* ≥ 420 nm 100 mW cm^−2^	Pure water	340	8.36 (420 nm)	[57]
C_3_N_5_/SubPc‐Br/ZnIn_2_S_4_	*λ* ≥ 400 nm 350 mW cm^−2^	tetracycline (20 mg L^−1^)	549	3.25 (400 nm)	[35]
C_3_N_4_/C_3_N_5_	*λ* ≥ 400 nm 350 mW cm^−2^	tetracycline (20 mg L^−1^)	446	—	[58]
DT2TA‐TAPB	*λ* ≥ 420 nm	10% ethanol	885	3.3 (380 nm)	[59]
TAPT‐TFPA COFs@Pd ICs	1.5 G solar intensity	10% ethanol	2143	6.5 (400 nm)	[60]
TpAP	*λ* ≥ 420 nm	10% ethanol	2343	3.18 (420 nm)	[61]

Recycling tests (Figure 2k) revealed only a slight decrease in both H_2_O_2_ production and RhB degradation after five consecutive cycles, demonstrating good operational stability. Further structural and compositional characterization via SEM‐EDS mapping (Figure S11, Supporting Information) confirmed the homogeneous distribution of C, N, O, and B elements on CSC(50), while XRD patterns (Figure S12, Supporting Information) showed no significant structural changes or phase degradation after cycling, confirming its robust stability. To further evaluate the long‐term durability, especially regarding the stability of the molecular SubPc component against potential hydrolysis and photobleaching, an extended 24 h continuous test was conducted in pure water. As shown in Figure S13 (Supporting Information), the catalyst maintained sustained H_2_O_2_ production throughout this prolonged period, preserving approximately 71% of its initial production efficiency. More importantly, comprehensive characterization of the postreaction catalyst confirmed its structural integrity: XPS spectra (Figure S13, Supporting Information) clearly showed the characteristic peaks corresponding to the amide bond and the SubPc macrocycle, confirming the stability of these key chemical bonds and indicating no significant chemical degradation, while elemental mapping (Figure S14, Supporting Information) showed a homogeneous distribution of boron without detectable leaching or aggregation.

The synergistic photocatalytic performance of CSC(50) for simultaneous H_2_O_2_ production and RhB degradation was further evaluated in solutions prepared with pure water, tap water, and seawater. The results indicated that the performance decreased by approximately 30% in tap water and 60% in seawater compared to that in pure water. This decline can be attributed to the presence of common coexisting ions in tap water and seawater—such as Cl^−^, CO_3_^2−^, Ca^2+^, and Mg^2^⁺—which may adsorb onto the active sites of the catalyst, thereby hindering the accessibility and reaction of pollutant molecules and O_2_ [18]. Additionally, high ionic strength could adversely affect interfacial charge transfer and the stability of reactive oxygen species (e.g., ^•^O_2_^−^).

### 2.3 Photocatalytic Mechanism Study

**FIGURE 3:**
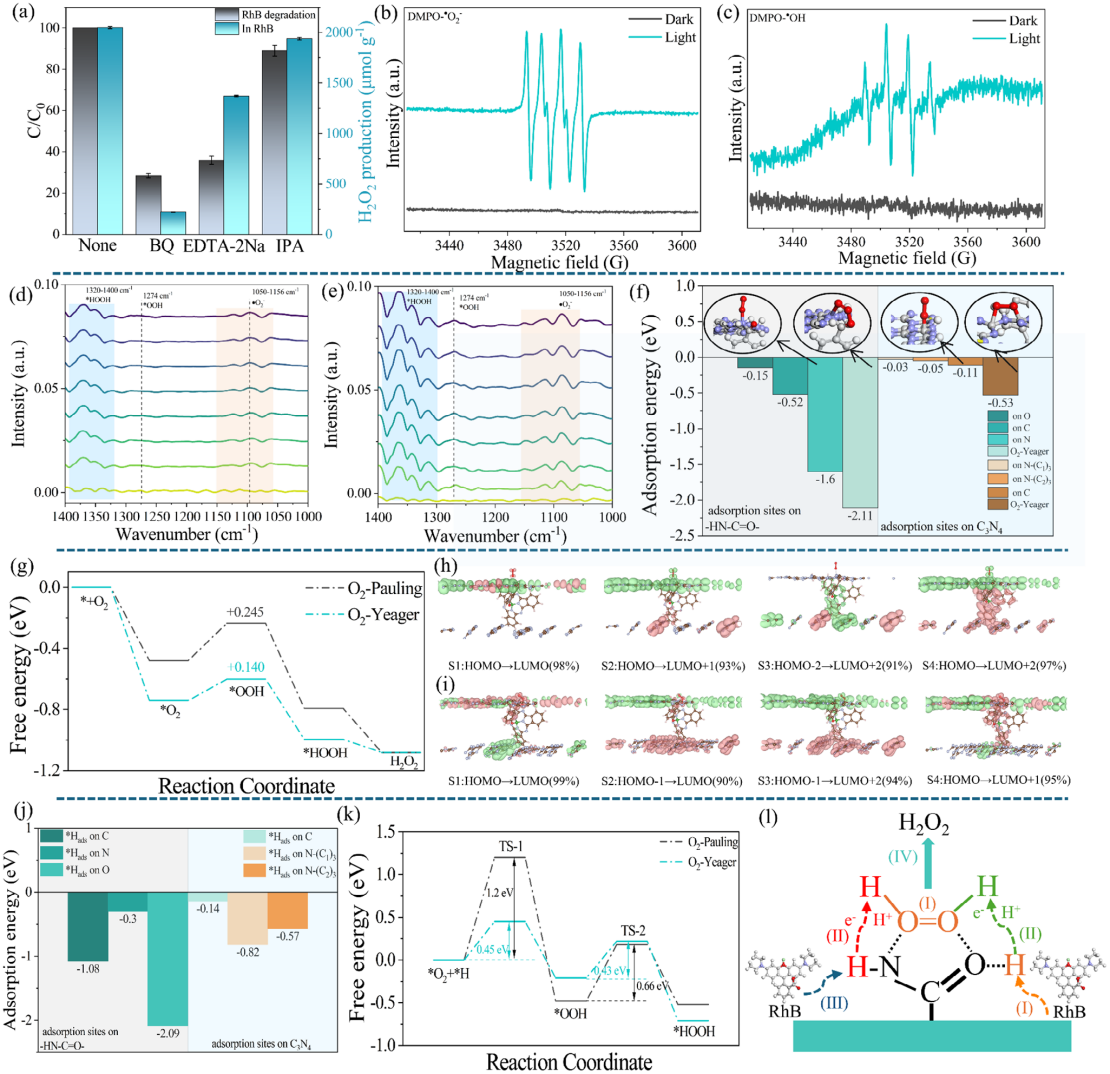
(a) Free‐radical trapping experiments, EPR spectra of (b) DMPO–•O_2_ and (c) DMPO–•OH recorded in the dark and under visible‐light irradiation; in situ DRIFT spectra acquired (d) during photocatalytic H_2_O_2_ production and (e) during the concomitant degradation of RhB; (f) O_2_ adsorption energies on different sites of CSC(50); (g) free‐energy profiles for O_2_ reduction to H_2_O_2_ on Yeager‐type and Pauling‐type models; TDDFT‐computed electron–hole distributions for the S_1_–S_4_ excited states of (h) Pauling‐type and (i) Yeager‐type models (pink = excited electron, green = excited hole); (j) *Hads adsorption energies on different sites of CSC(50); (k) energy barriers for *O_2_ → *OOH → *HOOH; (l) catalytic cycle of the one‐step 2e^−^ direct ORR mechanism over the CSC(50) system.

The band alignment plays a critical role in governing electron transfer across heterojunction interfaces. Precise modulation of the interfacial band structure is essential for optimizing the efficiency of photogenerated charge carrier separation and transfer. In this study, the flat‐band potentials (*E*_fb_) of C_3_N_4_, SubPc‐1, and C_3_N_5_ were systematically characterized via Mott–Schottky analysis (Figures S15–S17, Supporting Information). The measured *E*_fb_ values were determined to be −1.23, −0.67, and −0.24 V, respectively. According to classical semiconductor theory, the conduction band minimum (*E*_CB_) of an n‐type semiconductor is typically approximately 0.2 V more negative than its *E*_fb_. Thus, the *E*_CB_ values of C_3_N_4_, SubPc‐1, and C_3_N_5_ were estimated to be −1.43, −0.87, and −0.44 V (vs. NHE: −1.19, −0.63, and −0.20 V, respectively). The valence band maximum (*E*_VB_) was subsequently calculated using the relation *E*_VB_ = *E*_CB_ + *E*_g_, yielding values of 1.18, 1.28, and 1.77 V (vs. NHE), respectively. Such an arrangement serves as a prerequisite for forming an anisotropic dual S‐scheme heterojunction, thereby providing a solid foundation for efficient subsequent charge separation. To elucidate the underlying mechanism of the concurrent H_2_O_2_ production and RhB degradation, radical trapping experiments were performed on the CSC(50) system (Figure 3a). Specific quenchers, namely benzoquinone (BQ) for •O_2_^−^, disodium ethylenediaminetetraacetate (EDTA‐2Na) for h⁺, and IPA for •OH, were introduced. The findings clearly identify h⁺ and •O_2_^−^ as the principal reactive species driving RhB degradation. Furthermore, •O_2_^−^ is established as a critical intermediate for H_2_O_2_ formation, whereas •OH exerts only a marginal effect on both processes. This confirms that the CSC(50) composite primarily produces H_2_O_2_ via an indirect oxygen reduction pathway with ^•^O_2_^−^ as the key intermediate, though a direct 2‐electron oxygen reduction reaction (ORR_in_) may also occur to some extent, as evidenced by the residual H_2_O_2_ production even after BQ addition. Moreover, the dominant 2e^−^ pathway was quantitatively verified by rotating disk electrode (RDE) measurements. Koutecky–Levich analysis yielded an electron transfer number (*n*) of 1.80–2.03 for CSC(50), unequivocally confirming its high selectivity toward H_2_O_2_ production (Figures S18 and S19, Supporting Information). Electron paramagnetic resonance (EPR) spectroscopy further revealed a significantly enhanced signal for the DMPO‐^•^O_2_^−^ adduct in the CSC(50) composite (Figure 3b), indicating a higher concentration and greater generation capability of ^•^O_2_^−^, which contributes to the improved H_2_O_2_ yield. Although the signal for DMPO‐^•^OH was weak (Figure 3c), the valence band maximum (VBM) of C_3_N_5_ is considerably lower than the oxidation potential of H_2_O/^•^OH (+2.31 V vs. NHE). This thermodynamic constraint inhibits hole‐mediated water oxidation and subsequent ^•^OH generation. Therefore, we propose that the excess ^•^O_2_^−^ radicals in CSC(50) may react with H⁺ to form ^•^OH.

To elucidate the surface mechanism of H_2_O_2_ formation, in situ diffuse reflectance infrared Fourier transform spectroscopy (DRIFTS) was utilized. This technique enabled direct observation of interfacial reactions under a continuous O_2_ flow, allowing for a comparative analysis between environments of pure water vapor and RhB‐laden water vapor. As shown in Figure 3d,e, with prolonged irradiation time, the characteristic infrared absorption peaks of three key intermediates gradually intensified: the signal at 1274 cm^−1^ attributed to the O─H deformation vibration of adsorbed *OOH, the absorption band in the range of 1050–1156 cm^−1^ originating from the O─O stretching vibration of surface superoxide species (such as adsorbed O_2_^−^), and the O─H bending vibration mode corresponding to surface‐adsorbed hydrogen peroxide (HOOH) in the range of 1320–1400 cm^−1^. These results directly confirm the continuous generation and accumulation of H_2_O_2_ and its reaction intermediates on the surface of CSC(50) under light irradiation. In terms of signal intensity, the absorption peak of *HOOH is significantly stronger than those of *OOH and *O_2_^−^‐related species, indicating that the formation of H_2_O_2_ in this catalytic system may involve both the direct 2e^−^ ORR and an indirect reduction mechanism mediated by surface superoxide species. Notably, in the RhB‐containing water vapor environment, the infrared signal intensities of all intermediates were significantly higher than those under pure water vapor conditions, demonstrating that the presence of RhB markedly promotes the generation of H_2_O_2_. This enhancement is mechanistically linked to the dual function of RhB, which acts concurrently as an efficient hole scavenger and an external electron donor. Its role significantly suppresses charge recombination, thereby liberating a greater flux of electrons to drive the oxygen reduction reaction. This concerted action synergistically facilitates the multipath conversion of O_2_ to H_2_O_2_.

To further elucidate the photocatalytic generation mechanism of H_2_O_2_, we employed density functional theory (DFT) and time‐dependent DFT (TDDFT) calculations. Since oxygen adsorption is the first step of the oxygen reduction reaction (ORR), we systematically evaluated the possible adsorption sites of O_2_ on the CSC(50) surface. Owing to the more negative conduction band position of C_3_N_4_, the ORR primarily occurs on the C_3_N_4_ side. As shown in Figure 3f, we comprehensively examined the adsorption energies of O_2_ on both the C_3_N_4_ region and the amide bond connected to SubPc‐1, considering both Pauling‐type and Yeager‐type adsorption modes. The results indicate that O_2_ is most readily adsorbed in a Yeager‐type manner on the C and N atoms of the amide bond, with an adsorption energy of −2.11 eV; Pauling‐type adsorption of O_2_ on the N site of the amide bond is slightly weaker, with an adsorption energy of −1.6 eV. In comparison, the C_3_N_4_ surface exhibits relatively poor O_2_ adsorption capability. Differential charge density analysis reveals that in the Pauling‐type O_2_ adsorption configuration on the amide bond, O_2_ resides in an electron‐enriched region (Figure S20, Supporting Information), implying that under excited states, O_2_ in this configuration is more prone to lose electrons, which is unfavorable for the ORR. In contrast, in the Yeager‐type adsorption mode, O_2_ is located in an electron‐deficient region (Figure S21, Supporting Information), making it more likely to accept electrons in the excited state. This electronic distribution feature is also reflected in the density of states (DOS) diagram: the projected density of states (PDOS) of O_2_ in the Yeager‐type adsorption mode is closer to the bottom of the conduction band, further indicating that this adsorption configuration is more conducive to electron capture(Figure S22, Supporting Information). Furthermore, O_2_ temperature‐programmed desorption (TPD‐O_2_) results (Figure S23, Supporting Information) demonstrate that CSC(50) exhibits a significantly enhanced O_2_ adsorption capacity within the temperature range of 230–320°C compared to C_3_N_4_ and C_3_N_5_, further confirming that the formation of the amide bond between SubPc‐1 and C_3_N_4_ effectively promotes O_2_ adsorption. Figure 3g displays the DFT‐calculated free energy pathway for the O_2_→H_2_O_2_ conversion (the optimized structures of the intermediates are shown in Figure S24 in the Supporting Information), comparing the Pauling‐type and Yeager‐type O_2_ adsorption modes at the amide bond site of CSC(50). Thermodynamic analysis reveals a distinct advantage for the Yeager‐type configuration, where the strengthened O_2_ adsorption facilitates a more favorable proton–electron transfer step (Δ*G* = +0.140 eV) to form the critical *OOH intermediate. Conversely, the Pauling‐type mode suffers from a larger reaction barrier (Δ*G* = +0.245 eV), primarily due to a substantial spatial separation of ≈3.2 Å between the adsorbed O_2_ and the catalytic site that diminishes essential orbital overlap. This inference is quantitatively substantiated by Crystal Orbital Hamilton Population (COHP) analysis, which reveals a significantly weaker bonding interaction (ICOHP = −1.4 eV) for the Pauling‐type adsorption compared to the Yeager‐type configuration (average ICOHP = −3.92 eV) (Figure S25, Supporting Information). This geometric mismatch effectively impedes the direct hydrogenation of *O_2_. The superior performance of the Yeager‐type adsorption stems from its synergistic optimization of electrostatic and orbital interactions, thereby establishing a highly efficient pathway for the photocatalytic O_2_‐to‐H_2_O_2_ conversion. This fundamental mechanistic distinction provides a compelling explanation for the 8.95‐fold activity enhancement observed experimentally, underscoring the pivotal importance of atomic‐level spatial and electronic design in overcoming kinetic barriers in artificial photosynthesis. Subsequent TDDFT calculations further probed the excited‐state electron–hole distributions for both O_2_ adsorption modes on the amide bond. As shown in Figure 3h,i, yellow and pink regions represent excited‐state electrons and holes, respectively. In the Pauling‐type mode, only the S_1_ excited state exhibits electron accumulation on O_2_. In contrast, for the Yeager‐type mode, electron density is observed on O_2_ across the S_1_, S_2_, and S_4_ excited states. This result indicates that the Yeager‐type O_2_ adsorption mode is more prone to capture electrons, thereby facilitating the ORR process.

Furthermore, we investigated the dynamic behavior of coexisting H_2_O, O_2_, and H species within the CSC system using molecular dynamics (MD) simulations. It is important to note that these classical MD simulations primarily provide insights into the preferential accumulation and diffusion behavior of reactants at the heterojunction interface, but they do not capture the quantum mechanical aspects of the catalytic reactions, such as electron transfer and bond formation/breaking, which were explicitly addressed by our DFT and TDDFT calculations. With this scope in mind, snapshots from the equilibrated dynamics trajectory (Figure S26a–c, Supporting Information) reveal a noticeable accumulation of O_2_ and H at the heterojunction interface over time. This observation is further confirmed by the corresponding concentration profiles (Figure S26d, Supporting Information), which show significantly higher local concentrations of O_2_ and H compared to H_2_O at the interface. This selective enrichment of reactants creates thermodynamically favorable conditions for H_2_O_2_ formation. The diffusion coefficients, calculated from the mean squared displacement (MSD) curves (Figure S26e, Supporting Information), indicate that H possesses the highest diffusivity (2.81 × 10^−^⁶ cm^2^ s^−1^), followed by H_2_O (1.54 × 10^−^⁶ cm^2^ s^−1^), while O_2_ exhibits the lowest diffusion rate (8.37 × 10^−^⁷ cm^2^ s^−1^). This trend aligns with the molecular sizes and interfacial interaction strengths of the respective species: the small atomic size of hydrogen grants it high mobility, whereas the larger molecular volume and potential interfacial adsorption of O_2_ restrict its diffusion. The differences in diffusivity subsequently influence the transport efficiency of reactants to the active sites, thereby modulating the surface reaction kinetics.

To further elucidate the 2e^−^ ORR mechanism on CSC(50), we comparatively investigated the *Hads‐mediated hydrogenation pathway of *O_2_. First, the adsorption energies of *Hads at multiple potential sites on the amide bond and the C_3_N_4_ surface were computationally evaluated (Figure 3j), and the optimized *Hads configurations at different sites are presented in Figure S27 (Supporting Information). The results indicate that the adsorption energy of *Hads is the lowest (−‐2.09 eV) at the oxygen atom of the amide bond, suggesting that this site exhibits the strongest adsorption capacity for *Hads and serves as the preferential active site for the hydrogenation reaction. On the contrary, the adsorption energies at three sites on the C_3_N_4_ surface are relatively small (−0.14 to −0.82 eV). To further investigate the electrophilic reaction active sites on the surfaces of C_3_N_4_, C_3_N_5_, and SubPc‑5, we also calculated the color‐filled maps and isosurfaces of the localized orbital locator π (LOL‐π) and the electrostatic potential (ESP) for the three monomeric materials [62]. As shown in Figure S28a (Supporting Information), the high LOL‐π value regions on the outer side of the three six‐membered rings in C_3_N_4_ are interconnected, forming a complete large ring that clearly reveals the main delocalization pathway of the π‐electrons (Figure S28a, Supporting Information). In contrast, C_3_N_5_ does not form a similar large ring structure, but its five‐membered C─N ring exhibits relatively high LOL‐π values (Figure S28b, Supporting Information). From the LOL‐π isosurface plots, it can be observed that SubPc‑5 possesses the largest π‐electron region, which corresponds to a potential electrophilic reaction active site (Figure S28c, Supporting Information). Furthermore, the ESP map indicates that the axial carboxyl oxygen atom of SubPc‑5 has the highest electron density (Figure S28d, Supporting Information), suggesting that this site is most favorable for hydrogen adsorption. This is consistent with the adsorption energy results obtained from *Hads calculations, which show the most negative adsorption energy at the amide‐bonded oxygen atom in the composite material. Figure 3k displays the transition state (TS) energy profile for the hydrogenation reaction of O_2_ (O_2_ + *Hads → *OOH). The optimized transition‐state structures are shown in Figure S29 (Supporting Information). In the Yeager‐type O_2_ adsorption configuration, the energy barrier for *O_2_ → TS (Δ*G*_TS‑1_) is 0.45 eV, lower than that of the Pauling‐type adsorption (1.2 eV), indicating that the Yeager‐type adsorption is more favorable for the hydrogenation of O_2_. Furthermore, the energy barrier for the conventional pathway (O_2_ + e^−^ + H_2_O → *OOH + OH^−^) (Δ*G*_TS‑2_ = 0.95 eV) is significantly higher than that of the *Hads‐mediated pathway, further highlighting the critical role of the *Hads mechanism in promoting O_2_ hydrogenation. In the second hydrogenation step (OOH + *Hads → *HOOH), the energy barrier for the Yeager‐type adsorption (Δ*G*_TS‑2_ = 0.43 eV) is also lower than that of the Pauling‐type (0.66 eV). Additionally, we investigated the pathway where the original N─H of the amide bond and the *Hads adsorbed at the O site synergistically hydrogenate O_2_, which exhibits an energy barrier of 0.46 eV (Figure S30, Supporting Information), suggesting the feasibility of a one‐step direct 2e^−^ ORR process. In summary, the 2e^−^ ORR process on CSC(50) can be described as follows: O_2_ is adsorbed in the Yeager‐type mode on the amide bond, while the O site of the amide bond captures protons released from RhB degradation to form *Hads. This is followed by a two‐step indirect 2e^−^ ORR process, where *Hads sequentially hydrogenates *O_2_ and *OOH to ultimately generate H_2_O_2_ (Figure S31, Supporting Information). Additionally, a direct 2e^−^ pathway exists (Figure 3l), in which *Hads and the N─H of the amide bond cooperatively hydrogenate *O_2_ to form *HOOH, which is subsequently converted to H_2_O_2_. The protons released from RhB decomposition can also replenish the hydrogen lost at the N site of the amide bond, thereby regenerating the active site. Thus, the CSC(50) system achieves efficient and multipath H_2_O_2_ synthesis through a coupled mechanism of electron synergy and multipath proton transfer, significantly enhancing the efficiency and adaptability of the catalytic reaction.

It is noteworthy that in the RhB‐containing system, the photocatalytic efficiency is further enhanced through an additional sensitization mechanism. Beyond its roles as a hole scavenger and proton donor, RhB functions as an effective photosensitizer. Under visible light irradiation, RhB molecules absorb photons and inject excited electrons into the conduction band of the catalyst, thereby substantially augmenting the photogenerated electron pool available for the oxygen reduction reaction. This sensitization effect creates a synergistic collaboration with the anisotropic dual S‐scheme heterojunction and the proton relay function of the amide bond, leading to significantly enhanced overall efficiency for concurrent H_2_O_2_ production and pollutant degradation. Furthermore, it is worth considering whether intermediates generated during RhB degradation might influence the ORR pathways. While the complex degradation process of RhB, involving sequential de‐ethylation and chromophore cleavage, could theoretically interact with oxygen reduction, several lines of evidence indicate that such interference does not dominate the reaction: (1) the consistent identification of *OOH and *O_2_^−^ as the key intermediates in both pure water and RhB systems; (2) the maintained high selectivity toward H_2_O_2_ throughout the reaction; and (3) the excellent agreement between experimental observations and theoretical calculations performed on the heterojunction catalyst surfaces. Therefore, while some participation of organic fragments in secondary reactions cannot be entirely ruled out, the primary ORR pathway proceeds predominantly through the well‐defined routes on the catalyst surface, with RhB degradation functioning mainly as an efficient hole scavenger and proton source rather than significantly altering the fundamental oxygen reduction mechanism.

### 2.4 Electron Transport Mechanism Across the Heterojunction Interface

**FIGURE 4:**
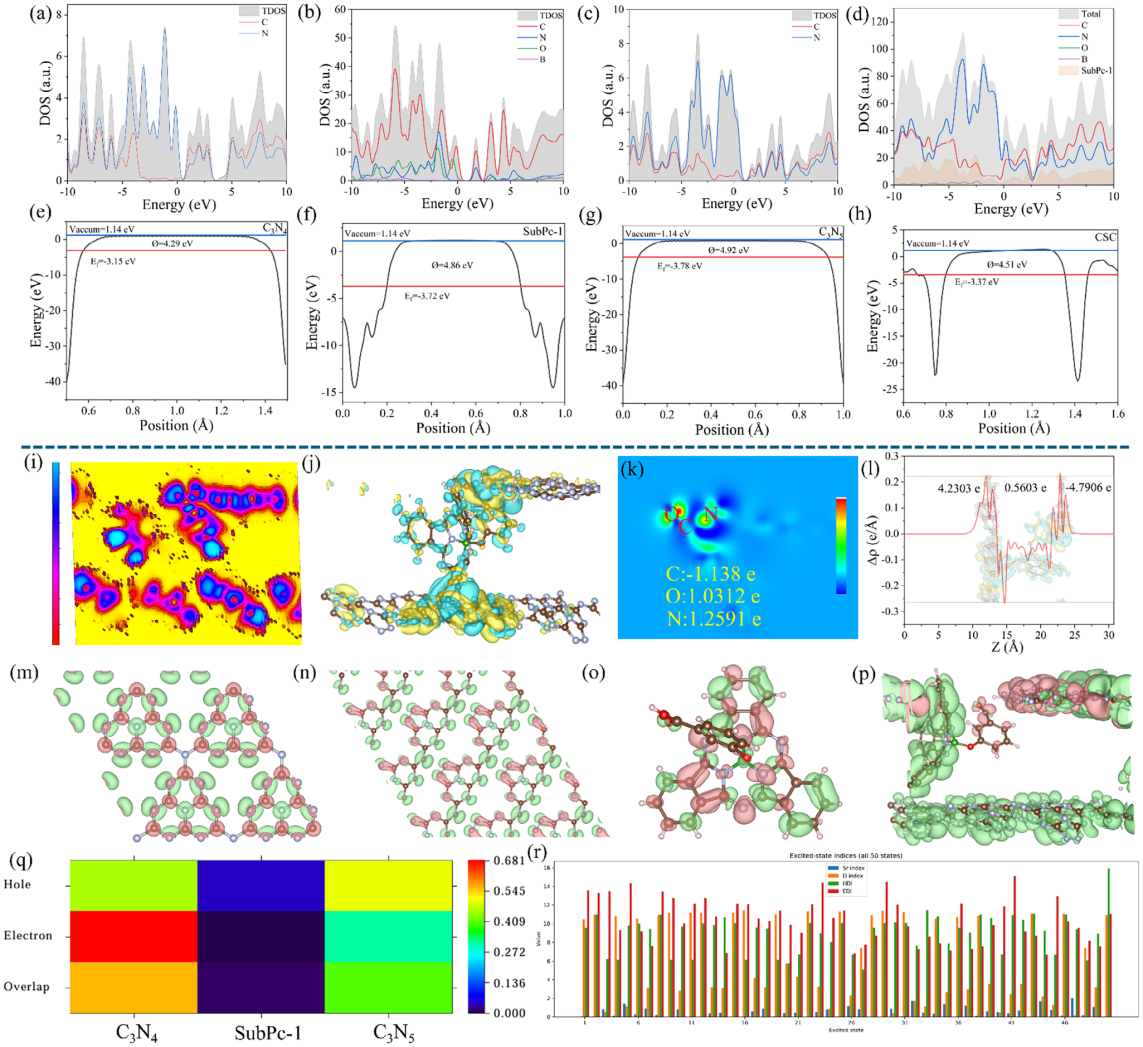
(a–d) Projected density of states (PDOS) of C_3_N_4_, C_3_N_5_, SubPc‐1, and the CSC heterojunction. (e–h) Electrostatic potential distribution along the *z*‐direction, (i) ELF, (j) 3D charge–density difference (isovalue ±0.001 e bohr^−^^3^): yellow, accumulation; cyan, depletion, (k) 2D charge–density difference, (l) Plane‐averaged charge–density difference along the *z*‐direction, (m–p) Electron–hole distribution maps of C_3_N_4_, SubPc‐1, C_3_N_5_, and the CSC composite (green: excited‐state holes; pink: excited‐state electrons), (q) Fragment‐resolved heat maps of electron, hole and their overlap for CSC, (r) Sr, D, HDI, and EDI indices for the 50 lowest excited states of CSC.

The charge separation dynamics within the CSC heterojunction were comprehensively probed using a multitechnique approach, integrating DFT, TDDFT, synchrotron‐based light‐illuminated XPS (SI‐XPS), and femtosecond transient absorption spectroscopy (fs‐TAS). Analysis of the DOS and PDOS elucidates that the VBM across C_3_N_4_, SubPc‐1, C_3_N_5_, and the composite CSC is principally constituted by C 2p and N 2p orbitals (Figure 4a–d). In contrast, the conduction band minimum (CBM) exhibits distinct compositional variations: it is dominated by N 2p states in C_3_N_4_, primarily composed of N 2p with a minor C 2p contribution in C_3_N_5_, and consists of hybridized C 2p and O 2p orbitals in SubPc‐1. In the CSC heterojunction, the introduction of SubPc‐1 leads to a shift in the CBM, where C 2p becomes the dominant orbital contribution, while that of N 2p is reduced. This modification in electronic structure indicates significant interfacial orbital hybridization and band renormalization between SubPc‐1 and the carbon nitride matrix. The enhanced C 2p contribution can be attributed to electronic coupling between the carbon‐rich units of SubPc‐1 and C_3_N_5_ or C_3_N_4_ at the heterojunction interface, which extends the π‐conjugation, reduces the effective mass of electrons, and facilitates the delocalization and migration of photogenerated electrons. This mechanism effectively improves interfacial charge separation efficiency, providing key theoretical insight into the superior photocatalytic performance of the CSC system. It also demonstrates that rational modulation of the electronic structure at heterojunction interfaces via molecular engineering is a viable strategy for designing high‐efficiency photocatalytic materials.

The difference in work function and Fermi level is one of the key experimental evidences for identifying the formation of an S‐scheme heterojunction. The electrostatic potential profiles (Figure 4e–h) indicate that C_3_N_5_ possesses a comparatively higher work function and lower‐lying Fermi level than SubPc‐1, which in turn exhibits analogs electronic characteristics relative to C_3_N_4_. This energy level gradient spontaneously drives electron transfer from components with higher Fermi levels to those with lower ones upon interfacial contact, until electronic equilibrium is established across the system. Consequently, a cascade of electron transfer occurs sequentially from C_3_N_4_ to SubPc‑1, and onward to C_3_N_5_. Furthermore, the electron localization function (ELF) analysis (Figure 4i) delineates the bonding nature within the CSC architecture, showing intense, localized electron density (blue regions) around the C═O and C─N bonds of the amide linkage. This pattern is characteristic of strong polar covalent bonds and provides definitive evidence for the successful formation of amide bonds. Further insight into the electron migration behavior at the heterojunction interface under the ground state is provided by the 3D charge density difference map (Figure 4j): cyan indicates electron depletion, while yellow indicates electron accumulation. A significant electron accumulation (yellow) is observed on the C_3_N_4_ side, whereas SubPc‐1 and C_3_N_5_ are dominated by electron depletion (cyan), indicating that electrons spontaneously transfer from C_3_N_4_ through SubPc‐1 to C_3_N_5_ even in the absence of light illumination. Combined 2D charge density difference and Bader charge analysis (Figure 4k) allow further examination of the charge distribution around the amide bond at the atomic scale: significant charge accumulation (yellow) is observed around O and N atoms, while the C atom is situated in a depletion region (cyan), quantitatively confirming that O and N atoms act as electron‐rich centers, forming potential nucleophilic sites that are prone to attract electrophilic species such as H⁺. The plane‐averaged charge density difference along the z‐axis (Figure 4l) provides an overall picture: C_3_N_4_ loses electrons overall, while SubPc‐1 and C_3_N_5_ gain electrons, clearly outlining the spontaneous electron transfer pathway in the heterojunction under the ground state. This establishes a built‐in electric field that facilitates the subsequent separation and transport of photogenerated carriers, laying the electronic structural foundation for efficient photocatalytic reactions.

To further investigate the electron transfer mechanism at the heterojunction interface under excited states, TDDFT calculations were performed. The absorption spectra of C_3_N_4_, SubPc‐1, C_3_N_5_, and the CSC composite are shown in Figures S32–S35 in the Supporting Information, respectively. The CSC composite exhibits a notably broadened absorption in the visible region, indicating enhanced light‐harvesting capability. We further conducted electron–hole analysis on the excited state corresponding to the strongest absorption peak. As shown in the electron–hole distributions in Figure 4m–p and the electron, hole, and transition density matrices in Figure S36 (Supporting Information), in pure C_3_N_4_, the peripheral N─(C_1_)_3_ regions exhibit localized excitation, where both electrons and holes are distributed in the same area, leading to a high probability of electron–hole recombination. The central N─(C_1_)_3_ regions contain excited‐state holes, while the C atoms host excited‐state electrons. No significant excited‐state electron–hole distribution is observed in the N(C_2_)_3_ regions. This excited‐state characteristic results in severe carrier recombination in C_3_N_4_, which is the fundamental reason for its low photocatalytic efficiency. In C_3_N_5_, due to broken structural symmetry, the excited‐state holes are mainly localized on N atoms, while the excited‐state electrons are concentrated on the C atoms of the five‐membered ring, indicating charge‐transfer excitation. This partially mitigates carrier recombination, although most C atoms still lack effective excited‐state distribution. In SubPc‐1, the excited state is primarily characterized by electron transfer from the peripheral C atoms to the C and N atoms in the five‐membered ring, also corresponding to charge‐transfer excitation. In the CSC heterojunction, the excited state exhibits typical charge‐transfer characteristics: electrons transfer from C_3_N_5_ to the SubPc‐1 macrocycle, then axially through SubPc‐1 to the C_3_N_4_ at the interface, while the distal part of C_3_N_4_ also contributes some electrons to the proximal region. Consequently, the excited‐state electrons are mainly distributed on the interfacial C_3_N_4_ side, while the holes are located on SubPc‐1 and C_3_N_5_. This mechanism not only facilitates in‐plane lateral electron transfer within C_3_N_4_ but also enables longitudinal electron migration across the CSC material. The spatial separation of holes and electrons significantly suppresses carrier recombination, thereby enhancing the efficiency of the photocatalytic reaction. To further quantify the excited‐state behavior, heat maps were used to illustrate the contributions of the three fragments—C_3_N_4_, SubPc‐1, and C_3_N_5_—in CSC to the excited‐state electrons, holes, and electron–hole overlap, as shown in Figure 3q. It is clearly observed that electrons are predominantly distributed on C_3_N_4_, while holes are mainly localized on SubPc‐1 and C_3_N_5_. In addition, heat maps depicting the contributions of individual atoms to electrons and holes across 50 excited states were generated (Figures S37–S40, Supporting Information). The results are generally consistent with the characteristics of the excited state corresponding to the strongest absorption peak. Furthermore, comparison of 50 excited‐state indices—including the overlap index of hole and electron distributions (Sr), the average hole–electron distance (D), the hole delocalization index (HDI), and the electron delocalization index (EDI)—between the monomers and the CSC composite revealed that CSC exhibits a larger D index and a smaller Sr index, indicating more significant spatial separation between holes and electrons and weaker carrier recombination [63]. However, the relatively high HDI and EDI values suggest that holes and electrons remain highly delocalized within their respective localized regions. This apparent contradiction—high degree of separation coupled with strong delocalization—can be rationalized by the interfacial charge transfer mechanism in the heterojunction: D and Sr reflect the separation effect between holes and electrons as two entities, improved by the interfacial electron migration from C_3_N_5_ to C_3_N_4_. In contrast, HDI and EDI reflect the extent of dispersion within the spatial distributions of the holes or electrons themselves. The large HDI and EDI values indicate that although the holes are primarily localized on the donor fragments (SubPc‐1/C_3_N_5_) and the electrons on the acceptor fragment (C_3_N_4_), within each fragment, the charges are not completely confined to a single atom but exhibit significant delocalization. This delocalization behavior facilitates the migration of carriers within the material, thereby maintaining high charge mobility while achieving effective charge separation, ultimately working synergistically to enhance the overall photocatalytic performance.

The interfacial charge transfer pathway was further elucidated by synchrotron‐irradiated X‐ray photoelectron spectroscopy (SI‐XPS). Analysis of the SI‐XPS data (Figure S41, Supporting Information) revealed that in the C_3_N_4_/SubPc‐1 heterojunction under dark conditions, the ─C≡N bond (from SubPc‐1) manifested a negative binding energy shift relative to its position in the pristine materials. Concurrently, the N─C≡N species (from C_3_N_4_) displayed a corresponding positive shift. This complementary shift behavior implies a net transfer of electron density from C_3_N_4_ to SubPc‐1 in the absence of light. Upon light illumination, the ─C≡N peak shifts positively, suggesting that electrons flow back from SubPc‐1 to C_3_N_4_. Similarly, in C_3_N_4_/SubPc‐1/C_3_N_5_, the ─C≡N binding energy shifts positively in the dark relative to that in C_3_N_4_/SubPc‐1, while the N─C≡N peak associated with C_3_N_5_ shifts negatively, revealing electron transfer from C_3_N_4_/SubPc‑1 to C_3_N_5_ under dark conditions. Under illumination, the ─C≡N peak shifts negatively and the N─C≡N peak shifts positively, indicating that electrons return from C_3_N_5_ to C_3_N_4_/SubPc‑1. Consistent with these results, the N 1s peak of C_3_N_4_/SubPc‑1/C_3_N_5_ exhibits a positive shift under light (Figure S42, Supporting Information). Notably, oxygen is exclusively present in SubPc‑1, and its SI‐XPS binding energy shifts are the most pronounced, clearly reflecting the electron flow involving SubPc‑1. Compared with pure SubPc‑1, the O 1s peak of C_3_N_4_/SubPc‑1 shifts negatively in the dark and positively under light (Figure S43, Supporting Information), confirming electron transfer from C_3_N_4_ to SubPc‑1 in the dark and the reverse under illumination. Likewise, relative to C_3_N_4_/SubPc‑1, the O 1s peak of C_3_N_4_/SubPc‑1/C_3_N_5_ shifts positively in the dark and negatively under light, indicating that electrons migrate from C_3_N_4_/SubPc‑1 to C_3_N_5_ in the dark, and flow in the opposite direction under light. Collectively, the SI‐XPS results of these characteristic elements provide solid evidence for an interfacial electron transfer pathway in which electrons move from C_3_N_4_ through SubPc‑1 to C_3_N_5_ in the dark, and revert under light illumination. This reversible electron flow under dark/light conditions effectively builds an internal electric field and promotes the spatial separation of photogenerated carriers, which is crucial for achieving high‐efficiency S‐scheme photocatalytic performance.

**FIGURE 5:**
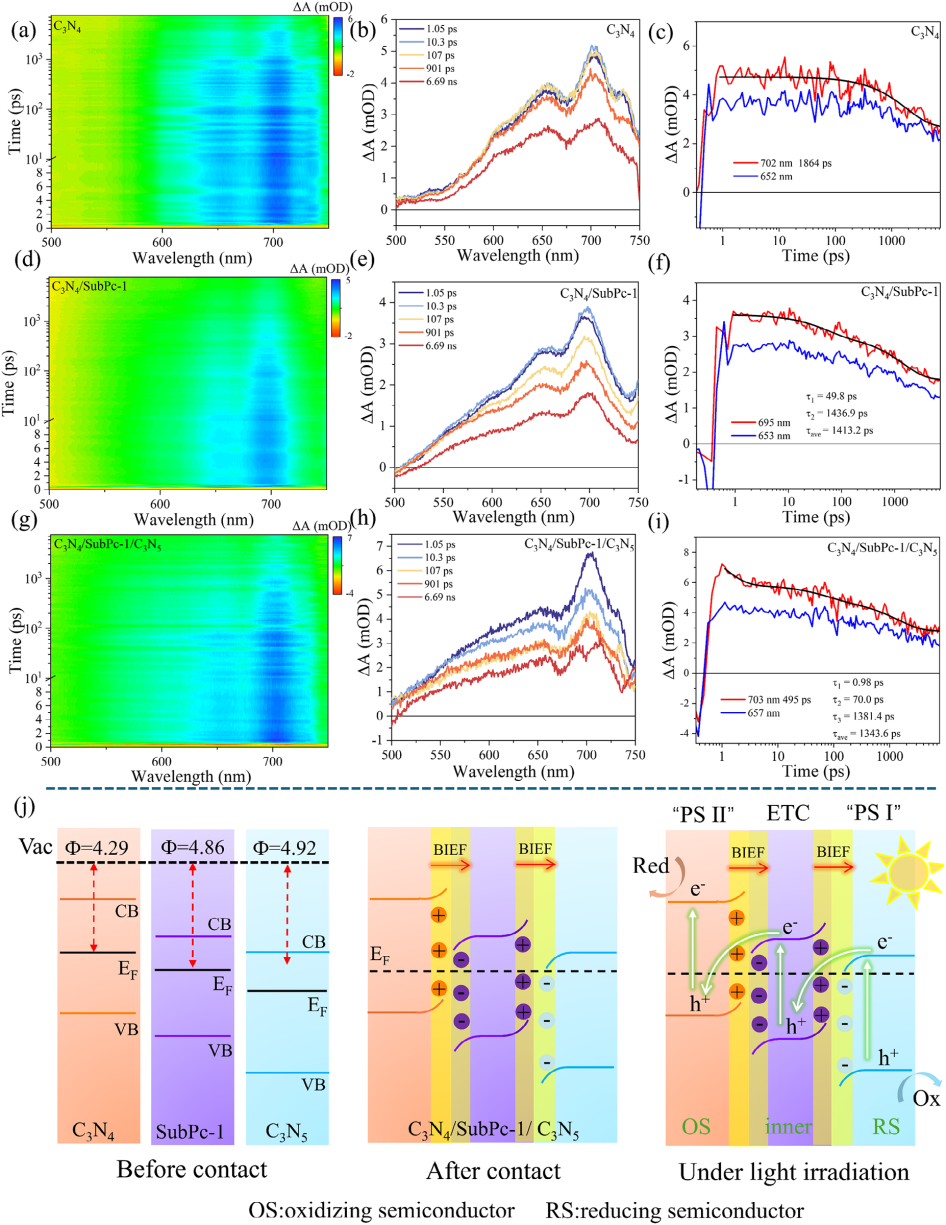
2D pseudo‐color transient absorption (TA) spectra recorded with 400 nm pump pulses for (a) C_3_N_4_, (d) C_3_N_4_/SubPc‐1, and (g) CSC(50). TA spectra at selected time delays for (b) C_3_N_4_, (e) C_3_N_4_/SubPc‐1, and (h) CSC(50). TA kinetics probed at (e) 470 nm and (f) 550 nm. TA kinetics measured at specific wavelengths for (c) C_3_N_4_, (f) C_3_N_4_/SubPc‐1, and (i) CSC(50). (j) Proposed anisotropic double‐S‐type electron‐transfer mechanism.

To gain deep insights into the photogenerated carrier dynamics within the C_3_N_4_/SubPc‑1/C_3_N_5_ composite system, a systematic investigation was conducted using fs‐TAS. Under 400 nm laser excitation, excited‐state absorption (ESA) signals were observed in the 500–750 nm range for pristine C_3_N_4_, the C_3_N_4_/SubPc‑1 binary heterojunction, and the C_3_N_4_/SubPc‑1/C_3_N_5_ ternary heterojunction. The 2D spectra (Figure 5a,d,g) and 1D kinetic profiles (Figure 5b,e,h) clearly delineate the relaxation pathways and recombination mechanisms of the charge carriers. The ESA spectrum of pristine C_3_N_4_ exhibits four characteristic peaks at 600, 652, 702, and 730 nm, with the most intense signal at 702 nm. A slight intensity increase of all ESA signals within the 1.05–10.3 ps time window is attributed to hot carrier cooling, followed by signal decay; the 730 nm peak vanishes after 901 ps. The kinetic data at 702 and 652 nm were fitted well with a single‐exponential decay model (lifetime of 1864 ps), reflecting the monomolecular recombination pathway of excitons in C_3_N_4_. The relatively long lifetime originates from the delocalization characteristic of the π‐conjugated system but also underscores its low charge separation efficiency. In the C_3_N_4_/SubPc‑1 binary heterojunction, the ESA signals undergo changes: the 600 nm signal persists, the 652 nm signal slightly shifts to 653 nm, the 702 nm signal blueshifts to 695 nm, and the 730 nm signal completely disappears. The peak at 695 nm is the most intense, though its intensity is lower than the 702 nm peak in pristine C_3_N_4_. The kinetics require a biexponential fit, yielding two lifetime components: *τ*_1_ = 49.8 ps and *τ*_2_ = 1436.9 ps, with an average lifetime of 1413.2 ps. The disappearance of the 730 nm signal indicates that the introduction of SubPc‑1 alters the excited‐state population of C_3_N_4_; this signal likely corresponds to a specific excitonic state in C_3_N_4_ that is quenched in the composite due to interfacial charge transfer. The shorter lifetime *τ*_1_ is assigned to the interfacial electron transfer process from C_3_N_4_ to SubPc‑1, while *τ*_2_ corresponds to the recombination of the separated charges. In the C_3_N_4_/SubPc‑1/C_3_N_5_ ternary heterojunction, the ESA signals evolve further: the 600 nm peak disappears, the 653 nm signal shifts to 657 nm, and the 695 nm signal redshifts back to 703 nm, with the latter being the most intense. The kinetics necessitate a triexponential fit, revealing three lifetime components: *τ*_1_ = 0.98 ps, *τ*_2_ = 70 ps, and *τ*_3_ = 1381.4 ps, with an average lifetime of 1343.6 ps. The disappearance of the 600 nm signal and the shift from 695 to 703 nm signify that the incorporation of C_3_N_5_ further modifies the interfacial energy level alignment and excited‐state properties. The ultrafast lifetime *τ*_1_ is attributed to the cascade electron transfer from C_3_N_5_, through SubPc‑1, to C_3_N_4_. *τ*_2_ is likely associated with trapping/detrapping processes at interface defects or interlayer charge redistribution, and *τ*_3_ represents the long‐lived separated state. The fitted lifetime parameters systematically demonstrate the evolution of charge carrier dynamics: from single‐exponential decay in pristine C_3_N_4_ (1864 ps) to biexponential decay in the C_3_N_4_/SubPc‑1 heterojunction (*τ*_1_ = 49.8 ps, *τ*_2_ = 1436.9 ps) and finally to triexponential decay in the CSC ternary heterojunction (*τ*_1_ = 0.98 ps, *τ*_2_ = 70 ps, *τ*_3_ = 1381.4 ps). The disappearance or shift of specific ESA signals in the composites fundamentally stems from the electronic structure coupling and band renormalization at the heterojunction interfaces, whereby the original excited‐state populations are supplanted by new interfacial charge‐transfer states. As the heterojunction structure is progressively built, the carrier dynamics evolve from a single recombination channel into a multipath mechanism encompassing ultrafast interfacial transfer, trap‐assisted relaxation, and long‐lived charge separation. This progression unveils the physical essence of how multivariate heterojunctions manipulate excited‐state evolution pathways to achieve highly efficient charge separation.

Based on theoretical calculations from DFT and TDDFT, combined with characterization results from SI‐XPS and fs‐TAS, the interfacial electron transfer in the CSC heterojunction follows an anisotropic double S‐scheme mechanism (Figure 5j). While direct experimental observation of directional charge migration in real space remains challenging, this mechanistic assignment is strongly supported by the collective evidence presented herein. Specifically, the three components—C_3_N_4_, SubPc‐1, and C_3_N_5_—exhibit sequentially increasing work functions and sequentially decreasing Fermi levels. When the three form a heterojunction, the difference in Fermi levels drives electrons to flow from C_3_N_4_ through SubPc‑1 to C_3_N_5_ until the Fermi levels of the system reach equilibrium. This process induces band renormalization: the energy bands of C_3_N_4_ bend upward, those of C_3_N_5_ bend downward, while the bands of SubPc‑1 bend downward at the interface with C_3_N_4_ and upward at the interface with C_3_N_5_, thereby forming two built‐in electric fields oriented in the same direction. Under light illumination, electrons at the heterojunction interface are driven by the combined effects of the built‐in electric fields, Coulomb interactions, and band bending to flow back from C_3_N_5_ through SubPc‑1 to C_3_N_4_. For electrons within SubPc‑1, the consistent direction of the electric field also promotes their directional migration toward C_3_N_4_. This transfer pathway preserves the strong oxidation capability of the valence band maximum of C_3_N_5_ and the strong reduction capability of the conduction band minimum of C_3_N_4_, while achieving efficient spatial separation of photogenerated carriers. Such an anisotropic double S‐scheme heterojunction, by virtue of its unique band structure and built‐in electric field configuration, demonstrates exceptional interfacial and bulk charge separation capabilities. It not only significantly enhances charge separation and transport efficiency but also enables an ultrafast electron transfer process (<1 ps), offering a new strategy for the design of highly efficient photocatalytic systems.

## 3 Conclusions

In conclusion, we have demonstrated a multidimensional biomimetic strategy to overcome the persistent kinetic mismatch between electron transfer and proton diffusion in photocatalytic H_2_O_2_ production. By constructing an imide‐bridged anisotropic dual S‐scheme heterojunction (C_3_N_4_/SubPc‐1/C_3_N_5_), we achieve synergistic regulation of charge and mass transport. The ingenious integration of an in‐plane nonplanar junction and an interlayer anisotropic S‐scheme pathway enables efficient lateral electron migration and vertical charge extraction, while the unique imide bridge serves as a dual channel for both electrons and protons. A combination of in situ spectroscopic techniques and multiscale theoretical simulations unequivocally reveals that the Yeager‐type O_2_ adsorption configuration on the amide bond lowers the energy barrier for the rate‐determining step, enabling efficient dual‐pathway (direct and indirect) 2e^−^ ORR. More importantly, the anisotropic dual S‐scheme mechanism, corroborated by DFT, TDDFT, SI‐XPS, and fs‐TAS, drives ultrafast (<1 ps) directional electron transfer and achieves spatial separation of photogenerated carriers with preserved strong redox potentials. This work transcends the conventional heterojunction engineering by simultaneously addressing the challenges of charge kinetics, proton supply, and reactant activation within a single integrated system. The proposed “interface and bridge” design principle offers a universal platform for optimizing other complex photocatalytic reactions involving multistep proton‐coupled electron transfers, such as CO_2_ reduction and N_2_ fixation, paving a new avenue for the development of high‐efficiency artificial photosynthetic systems.

## Conflicts of Interest

The authors declare no conflicts of interest.

## Supporting information




**Supporting File**: advs73579‐sup‐0001‐SuppMat.docx

## Data Availability

The data that support the findings of this study are available from the corresponding author upon reasonable request.
