# Supplementary material for: Ultrafast Electron Transfer Coupled with a Proton Relay in an Anisotropic Dual S‐Scheme Heterojunction for Overcoming Kinetics Mismatch in H2O2 Photosynthesis
Source: Adv Sci (Weinh). 2025 Dec 23;13(14):e22285. doi: 10.1002/advs.202522285 (PMC12970283; doi:10.1002/advs.202522285)
Supplement: Supplementary file 1 — Supporting File: advs73579‐sup‐0001‐SuppMat.docx [file ADVS-13-e22285-s001.docx]

**Supporting Information**

**Ultrafast Electron Transfer Coupled with a Proton Relay in an Anisotropic Dual S-Scheme Heterojunction for Overcoming Kinetics Mismatch in H₂O₂ Photosynthesis**

Bing Wang ^a^, Yao Liu ^a^, XiangBo Feng ^a*^, EnZhou Liu ^b^, YuZhen Zhao ^a*^, ZongCheng Miao ^a,c^, Zhuo Li ^b*^

*^a^ Shaanxi Key Laboratory of Liquid Crystal Polymer Intelligent Display, Technological Institute of Materials & Energy Science (TIMES), school of computer science, Xijing University, Xi’an, 710123, PR China.*

*^b^ School of Chemical Engineering, Northwest University, Xi’an, 710069, P.R. China*

*c School of Artificial Intelligence, Optics and Electronics (iOPEN), Northwestern Polytechnical University, Xi’an 710072, China*

*∗ Corresponding authors.*

*E-mail addresses: fengxiangbo@xjtu.edu.cn (XiangBo Feng)*, *zyz19870226@163.com (YuZhen Zhao)*, *lz@nwu.edu.cn (Zhuo Li)*

**Section 1. Materials and methods**

1.1 Materials

1.2 Material characterizations

**Section 2. Synthetic Procedures**

2.1 Synthetic of C_3_N_4_

2.2 Synthetic of H_12_SubPcB-OPhCOOH (SubPc-1)

2.3 Synthetic of C_3_N_5_

2.4 Synthetic of C_3_N_4_/SubPc-1/C_3_N_5_

2.5 Synthesis of physical mixture PM-CSC(50)

Section 3. Photocatalytic experiment and measurement

3.1 Photocatalytic production of H_2_O_2_

3.2 Photocatalytic H_2_O_2_ production coupled with degradation

Section 4. Theoretical calculations

4.1 DFT and TDDFT calculations

4.2 MD calculations

Section 5. Supplementary figures

**Section 1. Materials and methods**

**1.1** **Materials**

All chemical reagents employed in this investigation were analytically pure materials obtained from Sigma-Aldrich and applied directly without further purification treatment.

**1.2 Material characterizations**

Single-crystal X-ray structure determination of SubPc-1: Under a microscope, crystals exhibiting uniform texture, regular habit, suitable size and high transparency were carefully selected with a syringe needle and glued to a glass capillary at room temperature. Diffraction data were collected on a Bruker SMART APEX II CCD diffractometer equipped with a graphite-monochromated Mo-Ka source (λ = 0.71073 Å) using φ/ω scans over the appropriate θ range. The raw data were processed with multi-scan absorption and Lorentz-polarization corrections. The structure was solved by direct methods with SHELXS-97 and refined by full-matrix least-squares on F² using SHELXL-97. All non-hydrogen atoms were located from difference-Fourier maps and refined anisotropically; hydrogen atoms were placed in calculated positions and refined with a riding model and isotropic thermal parameters.

Optical Properties: UV-Vis absorption spectra were acquired using a Shimadzu UV-3600 spectrophotometer calibrated with BaSO₄ reference panels. Crystallinity Analysis: Phase identification was performed via X-ray diffraction (Rigaku D/max-IIIA, Cu Kα radiation). Molecular vibration modes were characterized by FTIR spectroscopy (Bruker Vector 002).

Microstructural Imaging: Morphological features and lattice structures were resolved through field-emission SEM (JEOL JSM-6390) and TEM (Talos F200X). Surface Chemistry: XPS and SI-XPS profiles were obtained with a Thermo Scientific ESCALAB 250Xi system (Al Kα source), referenced to the C 1s peak at 284.8 eV.

BET measurements were conducted on an ASAP 2460 analyzer (Micromeritics, USA), and contact-angle measurements were carried out using a Theta Lite instrument (Biolin Scientific, Sweden).

Electrochemical Profiling: CHI660E workstation configured with Pt counter electrode, Ag/AgCl reference, and 0.1 M Na_2_SO_4_ electrolyte recorded EIS and transient photocurrent responses.

Carrier Dynamics: Fluorescence spectra (λ_ex_=330 nm) and lifetime decay were monitored using Hitachi F7000 and F-4500 systems, respectively. Reactive Species Detection: EPR measurements (Bruker ELEXSYS II) identified ˙OH/˙O₂⁻ radicals using DMPO spin trapping.

Femtosecond Transient Absorption Measurements and Analysis: Femtosecond transient absorption (TA) spectroscopy was performed using a Helios pump-probe system (Ultrafast Systems), driven by a regenerative amplified laser source (Coherent). The Ti: sapphire laser amplifier (Astrella, Coherent) produced 800 nm pulses at a repetition rate of 1 kHz, with a pulse width of 100 fs and an energy output of 7 mJ per pulse. These pulses were split into two paths by a beam splitter. One path was introduced into an optical parametric amplifier (TOPAS, Coherent) to generate tunable pump pulses centered at 400 nm. The other was focused onto sapphire and YAG plates to produce a broadband white-light continuum used as probe beams, covering spectral ranges of 420–800 nm. A motorized optical delay line provided a controllable time delay between pump and probe pulses, with a maximum delay window of 8 ns. The pump beam, modulated at 500 Hz using a mechanical chopper, and the probe beam were co-focused onto the sample. The transmitted probe signal was collected and delivered to a fiber-coupled multichannel spectrometer equipped with a CCD detector for time-resolved spectral analysis.

**Section 2. Synthetic Procedures**

**2.1 Synthesis of C_3_N_4_**

Spread 5.0 g of melamine evenly in a lidded ceramic crucible, leave a 0.5 mm gap in the lid, place it in a muffle furnace, ramp to 550 °C at 2.5 °C min⁻¹ and hold for 4 h, cool naturally to room temperature, collect the yellow product and grind it into a fine powder, then stir in 0.1 M HNO₃ for 2 h, centrifuge-wash with deionized water until neutral, and vacuum-dry at 60 °C for 12 h to obtain pale yellow g-C₃N₄ powder.

**2.2 Synthetic of H_12_SubPcB-OPhCOOH (SubPc-1)**

Boron subphthalocyanine bromine (SubPc-0) was synthesized and purified following a previously reported literature procedure.^[1]^ A mixture of SubPc-0 (0.475 g, 1 mmol) and m-hydroxybenzoic acid (0.276 g, 2 mmol) was dissolved in 30 mL of toluene and stirred for two hours at room temperature under a nitrogen atmosphere. Subsequently, the resulting mixture was poured into an autoclave, the temperature was raised from room temperature to 130 ℃ for 10 h and kept for an additional 72 h. The reaction mixture was then cooled down to the ambient temperature at a speed of lowering 5 ℃ per hour. After cooling, the solvent was cautiously removed in a rotary evaporator. The remaining solid residue was thoroughly washed with methanol (3 × 10 mL) and dried at 70 ℃ for 8 h, which resulted in golden purple microcrystals.

**2.3 Synthetic of C_3_N_5_**

C_3_N_5_ was prepared using the one-step hot polymerization method.^[2]^ 4 g of 3AT powder was placed in a crucible and then heated at 500 ℃ for 3 h at a rate of 5.0 ℃/min in an oven. After cooling to room temperature, the resulting brown C_3_N_5_ was ground into powder for further use.

**2.4 Synthetic of C_3_N_4_/SubPc-1/C_3_N_5_**

First, 50 mg of SubPc-1 was dispersed in 100 mL of toluene and sonicated at room temperature for 10 min. Subsequently, the corresponding amounts of C₃N₄ were added to the dispersion to achieve SubPc-1:C₃N₄ mass ratios of 1:25, 1:50, 1:75, and 1:100. The mixtures were stirred and reacted at 120 °C for 4 h. Next, 50 mg of C₃N₅ catalyst was introduced into each system, and the reaction was continued at 120 °C for another 4 h. After completion, toluene was removed by rotary evaporation. The crude products were thoroughly washed with ultrapure water and methanol, followed by vacuum drying at 80 °C for 8 h. The resulting materials were labeled as CSC(25), CSC(50), CSC(75), and CSC(100), according to the initial mass ratios.

**2.5 Synthesis of physical mixture PM-CSC(50)**

The physical mixture control sample, denoted as PM-CSC(50), was prepared using a simple mechanical mixing procedure designed to mimic the composition of the covalent CSC(50) heterojunction while avoiding the formation of chemical bonds. Specifically, 50 mg of C₃N₄, 1 mg of SubPc-1 (to maintain the 1:50 mass ratio), and 50 mg of C₃N₅ were combined directly in an agate mortar. The powder mixture was thoroughly ground for 30 minutes to ensure homogeneous dispersion. This mechanical blending was performed at room temperature without the use of any solvent or thermal treatment, thereby precluding the possibility of an amidation reaction between the components. The resulting finely mixed powder was collected and used directly for subsequent characterization and photocatalytic tests.

**Section 3. Photocatalytic experiment and measurement**

**3.1 Photocatalytic production of H_2_O_2_**

In a typical run, 15 mg of photocatalyst was dispersed in 50 mL of de-ionized water and stirred for 30 min in the dark to achieve adsorption-desorption equilibrium. The reaction was then initiated by irradiating the suspension with a 300 W xenon lamp (400–780 nm, 350 mW cm^-2^) under ambient air. At selected intervals, an aliquot of the suspension was withdrawn, filtered, and analyzed for H₂O₂ by the iodometric method. A 1 mL reaction sample was mixed with a solution comprising 1 mL of 0.4 M potassium iodide (KI) aqueous solution and 1 mL of 0.1 M potassium hydrogen phthalate (C_8_H_5_KO_4_) aqueous solution, followed by a complete dark reaction for 30 min. Based on the chemical equation: H_2_O_2_ + 3I^−^ +2H^+^→I_3_^−^ +2H_2_O, the concentration of H_2_O_2_ can be accurately calculated in accordance with the concentration of triiodide anion (I_3_^−^ ). The concentration of I_3_^−^ can be determined by observing the strong absorption of I_3_^−^ at 350 nm in the UV–vis absorption spectrum. In addition, titration strategies involving ammonium oxalate and potassium permanganate were employed to accurately identify the concentration of commercial 30 % H_2_O_2_. The corresponding standard curves of H_2_O_2_ concentration using iodometry are presented in Figure S1.


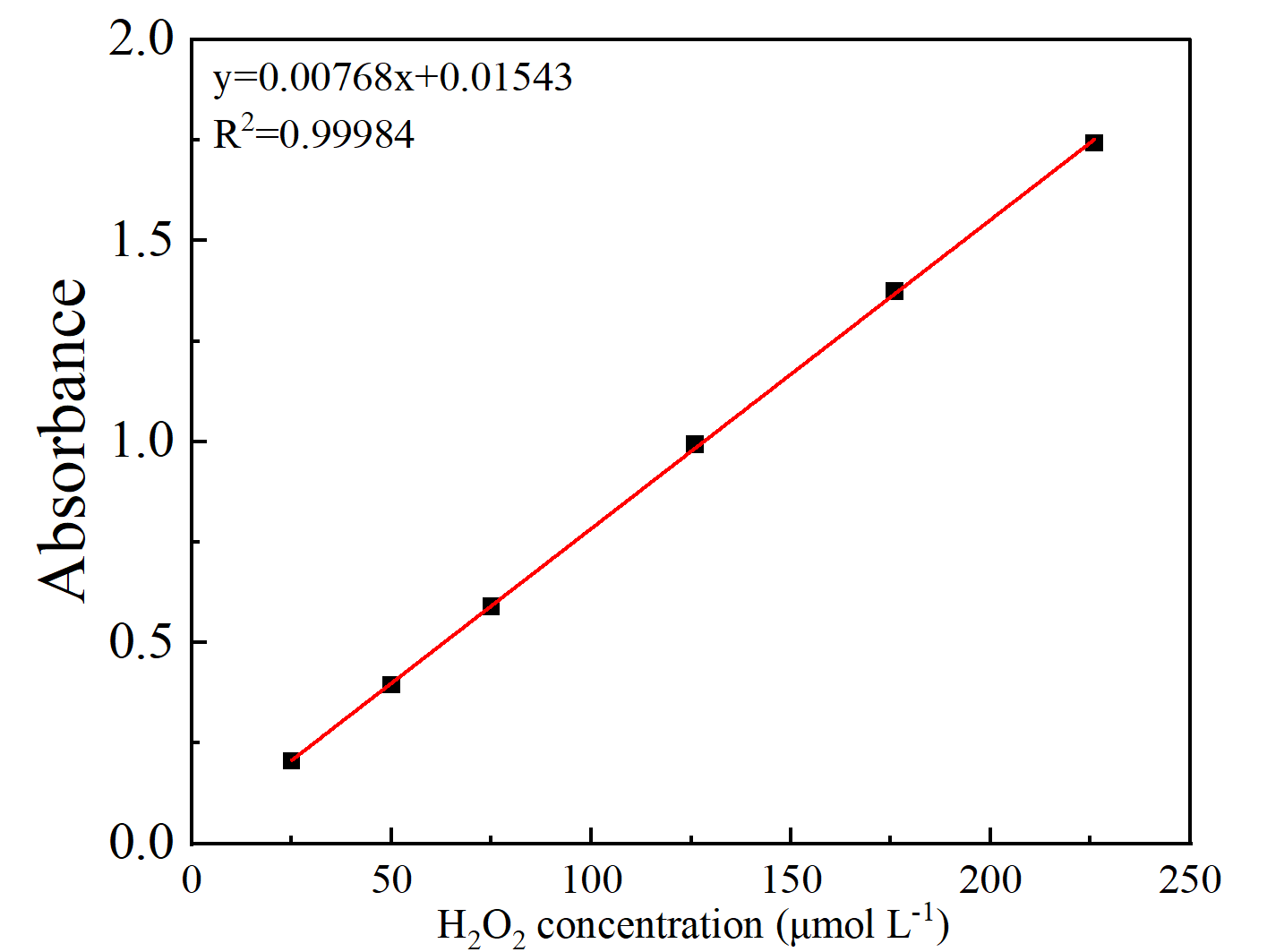


**Figure S1.** The corresponding standard curves of H_2_O_2_ concentration.

**3.2 Photocatalytic H_2_O_2_ production coupled with degradation**

In order to produce H_2_O_2_ and wastewater purification at the same time, the reaction conditions are the same as the above-mentioned H_2_O_2_ production process, except that the reaction solution is replaced by oxytetracycline (OTC, 20 mg L^−1^), tetracycline (TC, 20 mg L^−1^), bisphenol A (BPA, 20 mg L^−1^) and rhodamine B (RhB, 20 mg L^−1^) aqueous solutions. The concentration of OTC, TC, BPA and RhB contaminants was detected at wavelengths 356, 357, 278 and 552 nm, respectively, and the concentration of H_2_O_2_ was monitored by the iodine amount method.

The cyclic performance of a catalyst is a key indicator for evaluating its effectiveness. To this end, we conducted a series of experiments. First, after the first photodegradation process of the antibiotic, we filtered the solution and collected the resulting photocatalyst. Subsequently, the catalysts were repeatedly washed with deionized water and anhydrous ethanol, centrifuged, and dried in a vacuum oven at 60 °C for 6 hours for use in the next photocatalytic experiment. During the entire experiment, other conditions remained unchanged. After each cycle, we repeat the above steps until the multi-cycle experiment is completely finished.

**Apparent quantum yield (AQY) measurements:**

The AQY for H_2_O_2_ was measured under the irradiation of a 300 W Xe lamp equipped with different bandpass filters (400, 500, 550, and 600 nm). After ultrasonication and O_2_ bubbling, the photocatalytic reaction was conducted in RhB (50 mL) with photocatalyst (50 mg) at 25℃. The AQY is calculated by the following equation:^[3]^

$$\mathrm{AQY}\left( \% \right) = \frac{2M}{N_{\mathrm{photon}}} \times100 \% (Eq.1)$$

$$N_{\mathrm{photon}} = \frac{E_{\mathrm{total}}}{E_{\mathrm{photon}}} = \frac{ISt\lambda}{N_{A}\mathrm{hc}} (Eq.2)$$

M = yield of H_2_O_2_ (mol);

Na (Avogadro constant) = 6.02 × 10^23^ mol^−1^;

h (Planck constant) = 6.626 × 10^−34^ J·s = 4.136 × 10−15 eV s);

c (Speed of light) = 3 × 10^8^ m s^−1^;

S (Irradiation area) = 12.5 cm^2^;

I = the intensity of irradiation light (W cm^−2^);

t = the photoreaction time (s);

λ = the wavelength of the monochromatic light (m)

**Section 4. Theoretical calculations**

**4.1 DFT and TDDFT calculations**

DFT Simulations: ESP analyses of C₃N₄, SubPc-1, and C₃N₅ were conducted using DFT with the B3LYP hybrid functional, 6-31G(d) basis sets, and D3 dispersion corrections.^[4]^ All electronic-structure calculations (band structure, density of states, and work function) were performed with the plane-wave pseudopotential code VASP 5.2, using the GGA-PBE exchange-correlation functional.^[5]^ A plane-wave cutoff of 500 eV and Monkhorst-Pack k-meshes of 2 × 2 × 1 (structural relaxation) and 3 × 3 × 1 (electronic properties) were employed. Energy and force convergence criteria were set at 10^-4^ eV and 0.02 eV/Å, respectively. In addition, the projected COHP and the integrated COHP (ICOHP) were calculated using the LOBSTER code based on the plane-wave wavefunctions obtained from the VASP electronic-structure calculations. The Gibbs free energy (G) of each species (reactants, intermediates, products, and transition states) along the reaction pathway was evaluated as

$$G=E+ZPE-TS$$

where E, ZPE, and TS denote the DFT‑calculated total energy, zero‑point energy correction, and entropic contribution (at T = 298.15 K), respectively. Vibrational frequency calculations were performed on the optimized structures to obtain ZPE and entropy values, and the corresponding thermodynamic corrections were extracted using the VASPKIT code.^[6]^ Transition states (TSs) were located using the constrained Broyden‑based TS‑searching method^[7, 8]^ and the double‑ended surface walking (DESW) approach.^[9]^ All identified TSs were verified by frequency analysis to possess exactly one imaginary frequency

Excited-state properties of all materials were computed with CP2K, employing the hybrid PBE functional with D3 dispersion corrections and the DZVP-MOLOPT-SR-GTH basis set to obtain the lowest 50 excited states.^[10]^ The Multiwfn program was employed for post-processing excited-state analyses,^[11]^ including electron-hole distributions, absorption spectra, various exciton indices, and the contribution of each atom to the electron and hole of every excited state.

**4.2 MD calculations**

Molecular-dynamics (MD) simulations of O₂, H₂O and H on the surfaces of CSC composite were carried out with the Forcite module. Condensed-phase dynamics employed the UNIVERSAL force field, with atom-based Ewald summation (9.50 Å cutoff) for electrostatic and non-bonded interactions. A Nosé thermostat maintained the system at 298 K for 1000 ps with a 0.1 fs time step. Prior to production runs, full geometry optimization was performed. Prior to dynamics simulations, the system underwent geometric optimization.

**Section 5. Supplementary tables and figures**

**Table S1.** Crystallographic data and structure refinements for SubPc-1.

| **complex** | **SubPc-1** | | |
| --- | --- | --- | --- |
| formula | C_31_H_19_BN_6_O_4_ | *Z* | 2 |
| formula weight | 550.33 | *D/*g·cm^-3^ | 1.392 |
| *T*/K | 296(2) | *F*(000) | 568 |
| crystal system | Monoclinic | *μ*/mm^−1^ | 0.095 |
| space group | p21/n | data/restraints/Parameters | 5031/0/380 |
| *a*/Å | 9.047(3) | Goodness-of-fit on *F*^2^ | 1.030 |
| *b*/Å | 12.868(7) | *R_1_*^a^ [I >2σ(I)] | 0.0958 |
| *c*/Å | 12.993(4) | *wR_2_*^a^ [I >2σ(I)] | 0.2278 |
| *α*/° | 106.868(8) | *R_1_*^a^ (all data) | 0.2270 |
| *β*/° | 108.918(6) | *wR_2_*^a^ (all data) | 0.3042 |
| *γ*/° | 99.407(9) | Largest diff peak/hole (e·Å^-3^) | 0.851, -0.353 |
| ^a^*R*_1_ = Σ\|\|*F*_o_\| – \|*F*_c_\|\|/Σ\|*F*_o_\|; *wR*_2_=[Σ*w*(*F*_o_^2^–*F*_c_^2^)^2^/Σ*w*(*F*_o_^2^)^2^]^1/2^ | | |  |

**Table S2.** Selected bond distances (Å) and bond angles (°) for SubPc-1.

| **Complex** | **SubPc-1** | | |
| --- | --- | --- | --- |
| O(1)-B(1) | 1.446(8) | O(1)-C(25) | 1.375(7) |
| N(5)-B(1) | 1.530(8) | O(3)-C(31) | 1.295(8) |
| N(3)-B(1) | 1.477(9) | O(2)-C(31) | 1.258(9) |
| N(1)-B(1) | 1.487(8) | N(2)-C(8) | 1.338(7) |
| N(4)-C(16) | 1.319(7) | N(4)-C(17) | 1.353(7) |
| N(2)-C(9) | 1.343(7) | N(6)-C(1) | 1.360(7) |
| N(3)-B(1)-N(1) | 104.8(5) | C(25)-O(1)-B(1) | 128.2(5) |
| N(3)-B(1)-N(5) | 103.8(5) | O(1)-C(25)-C(30) | 115.4(6) |
| N(1)-B(1)-N(5) | 102.8(5) | O(2)-C(31)-O(3) | 119.1(8) |
| O(1)-B(1)-N(3) | 117.6(6) | O(2)-C(31)-C(27) | 121.8(7) |

**Table S3**. Fluorescence lifetimes of charge carriers for the as-prepared materials

| Sample | τ_1_ (ns); Rel% | τ_2_ (ns); Rel% | τ_av_ (ns) |
| --- | --- | --- | --- |
| C_3_N_4_ | 3.243; 80.48 | 22.36; 19.52 | 15.2 |
| C_3_N_5_ | 3.095; 70.56 | 21.185; 29.44 | 16.4 |
| SubPc-1 | 2.370; 85.50 | 21.846; 14.50 | 14.24 |
| CSC(25) | 2.251; 94.10 | 60.960; 5.9 | 39.19 |
| CSC(50) | 2.362; 89.72 | 37.555; 10.28 | 25.08 |
| CSC(75) | 2.472; 75.16 | 22.069; 24.84 | 17.10 |
| CSC(100) | 2.3736; 83.59 | 27.335; 16.41 | 19.68 |

**
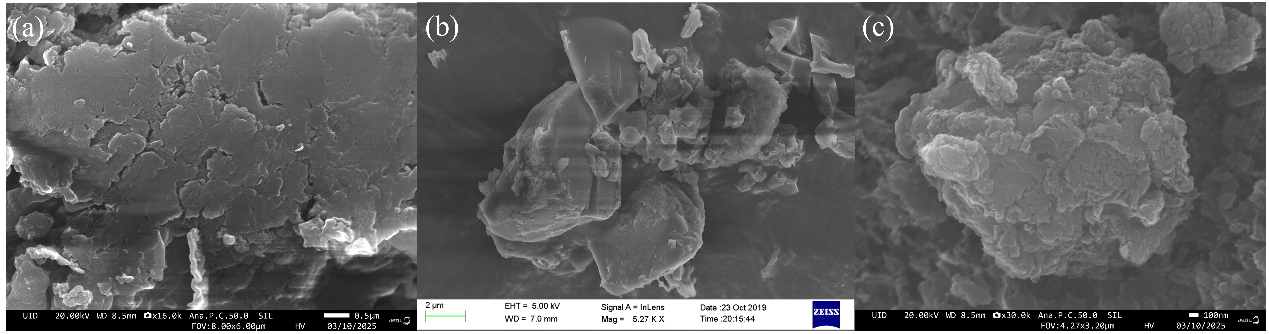
**

**Figure S2.** SEM images of (a) C3N4, (b) SubPc-1, and (c) C3N5.

**
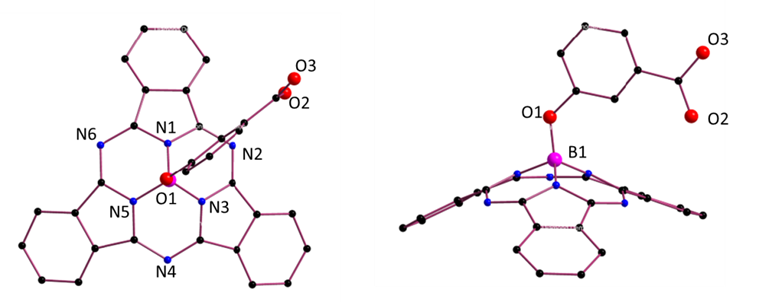
**

**Figure S3.** Face and side view of SubPc-1 single crystal structure.(C, black; N, blue; B, pink; Br, orange; H atom not shown).


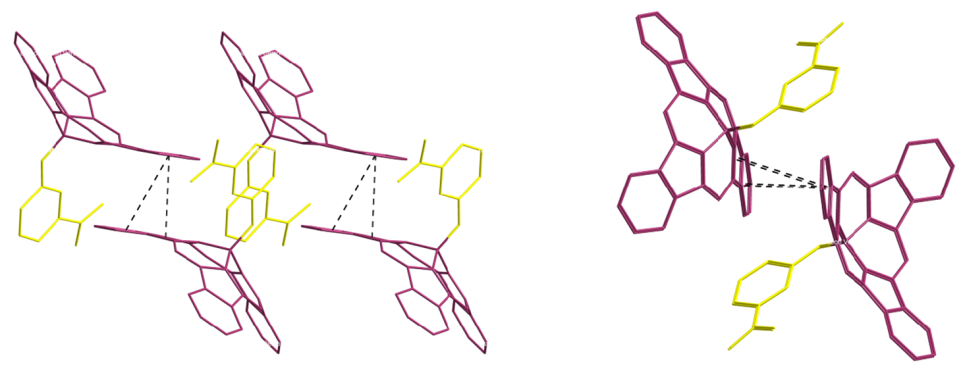


**Figure S4.** A section of the unit cell of SubPc-1(2) facing the ac plane(left) and a axis(right) showing the π-π extended stacking pathways.The BsubPc and phthalimide segments have been colored purple and yellow, respectively.

**
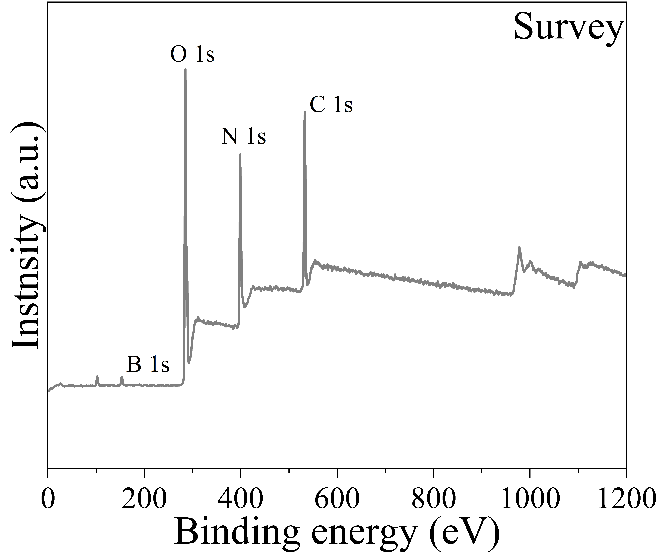
**

**Figure S5.** Survey XPS spectra of CSC(50).

**
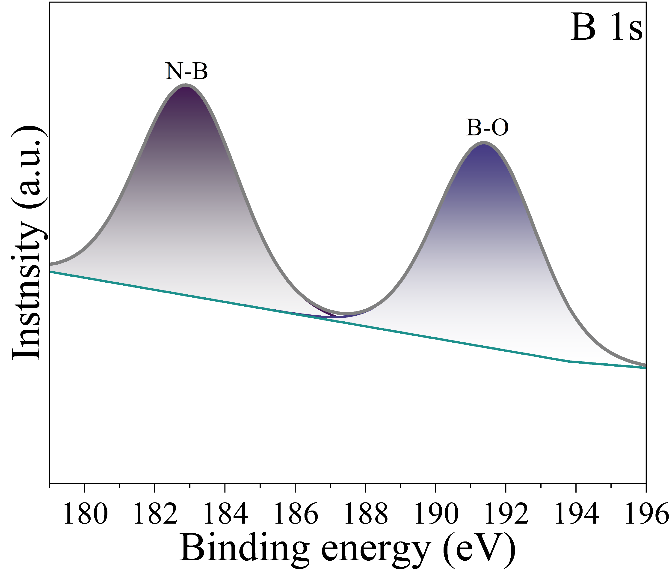
**

**Figure S6.** High-resolution B 1s XPS spectra of CSC(50).


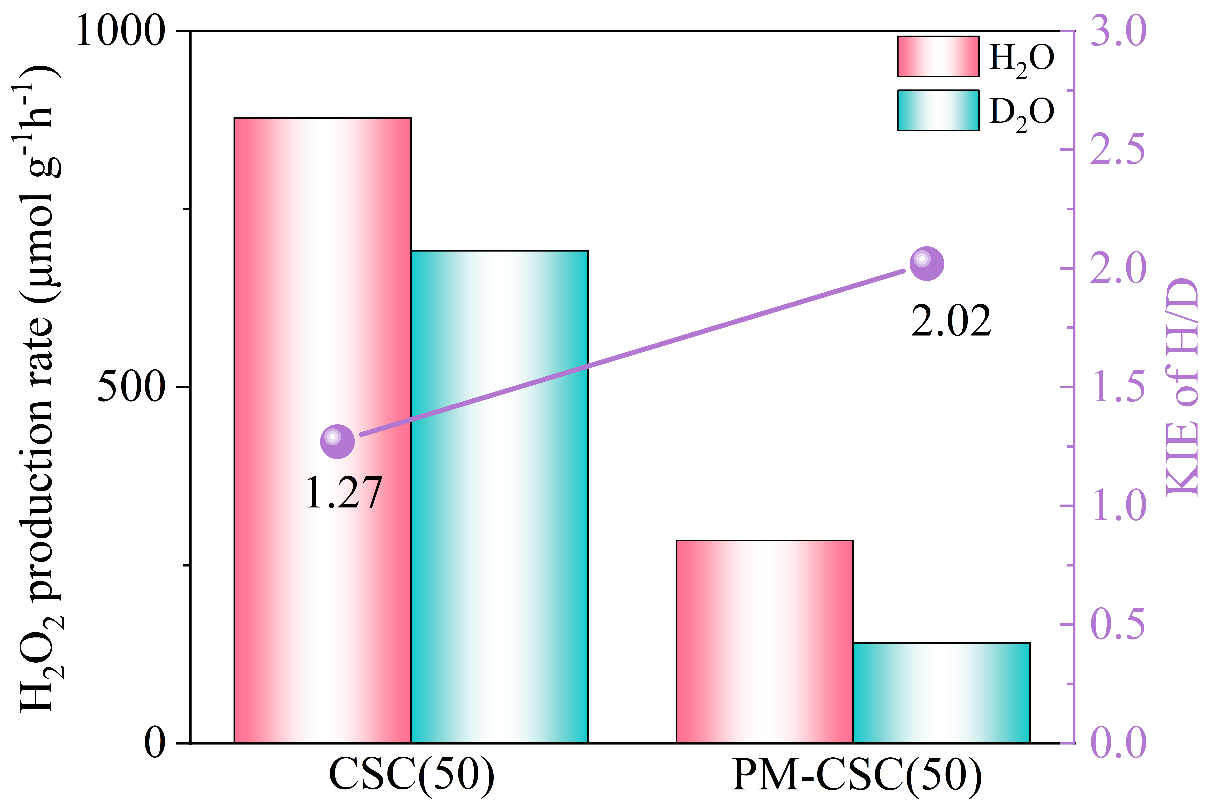


**Figure S7.** Photocatalytic H_2_O_2_ production rates of the covalent CSC(50) heterojunction and its physical mixture (PM-CSC(50)) control in H_2_O and D_2_O, and the corresponding calculated KIE values (RateH_2_O/RateD_2_O).


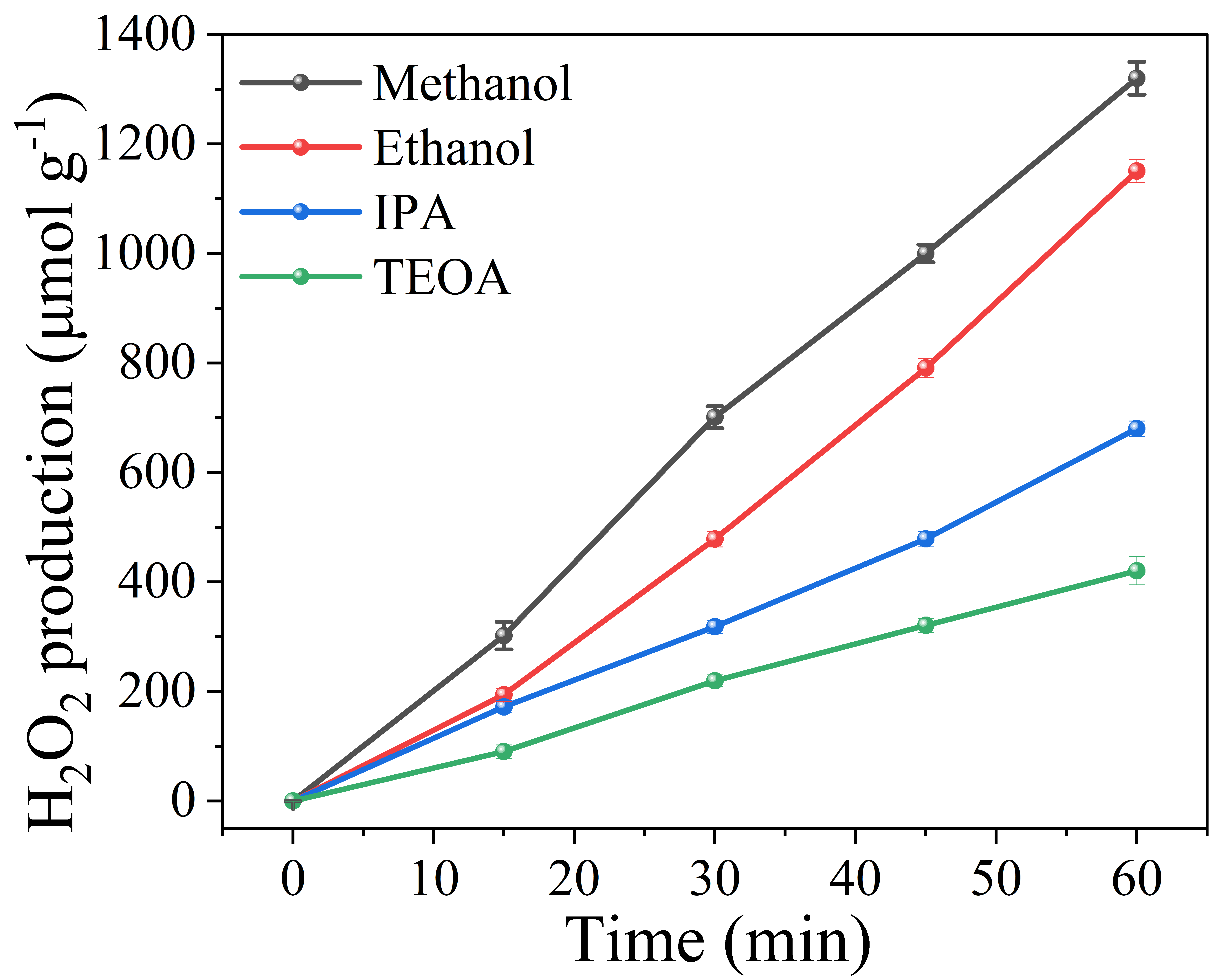


**Figure S8.** Photocatalytic H_2_O_2_ production over the CSC(50) catalyst with various non-absorbing sacrificial agents.


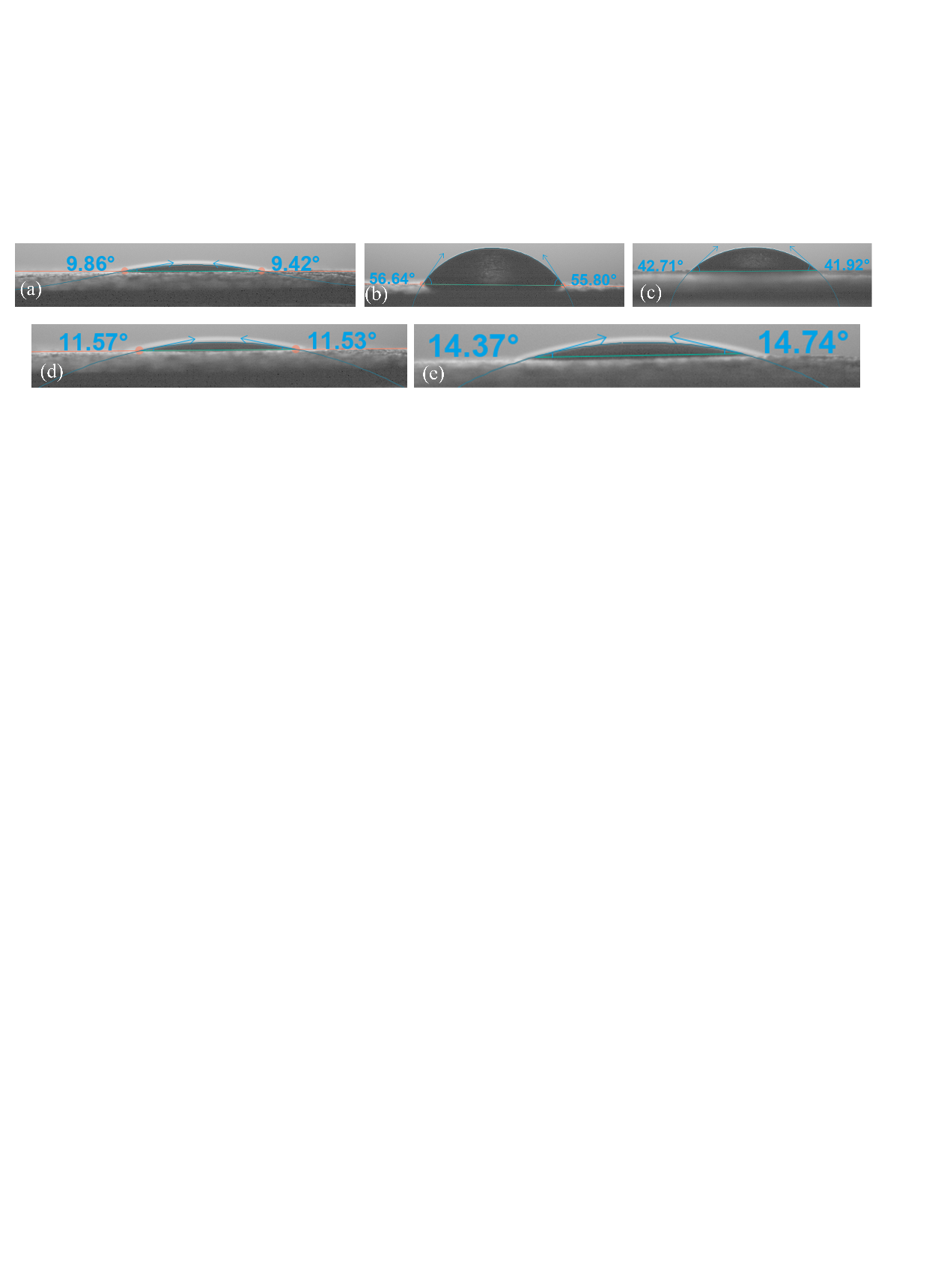


**Figure S9.** Contact-angle measurements of CSC(50) in a) H_2_O, b) OTC, c) TC, d) BPA, and e) RhB solutions.


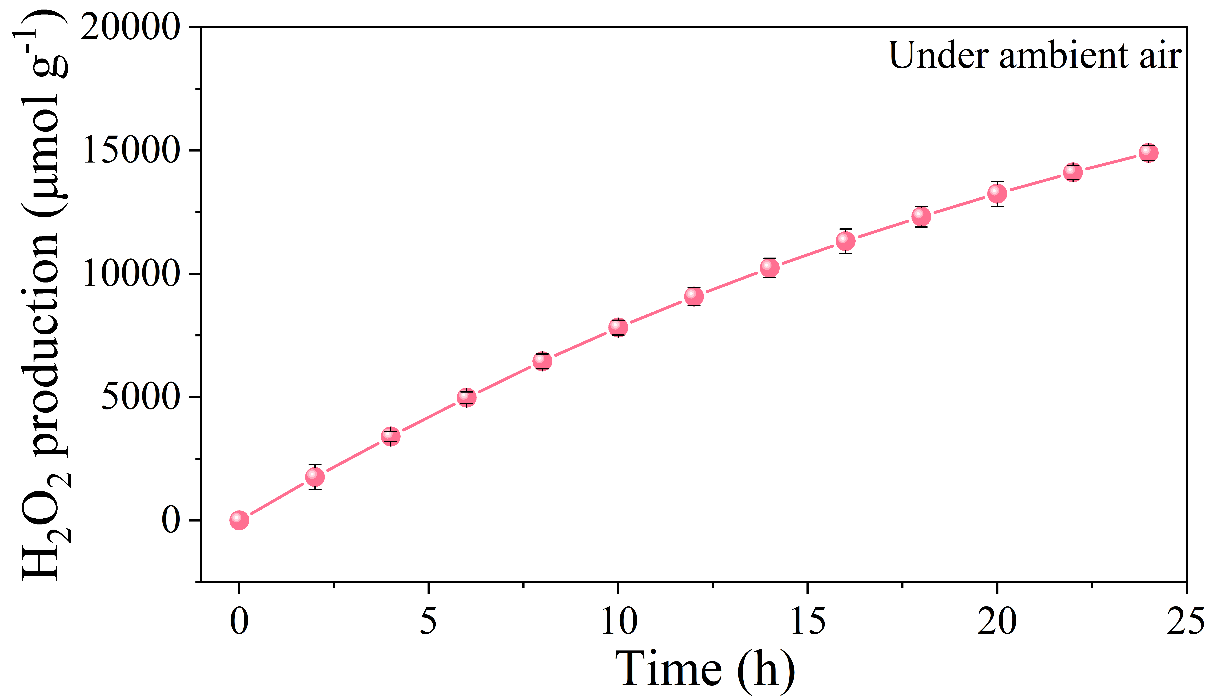


**Figure S10.** Time-dependent H_2_O_2_ accumulation profile during 24-hour continuous photocatalytic reaction in pure water.


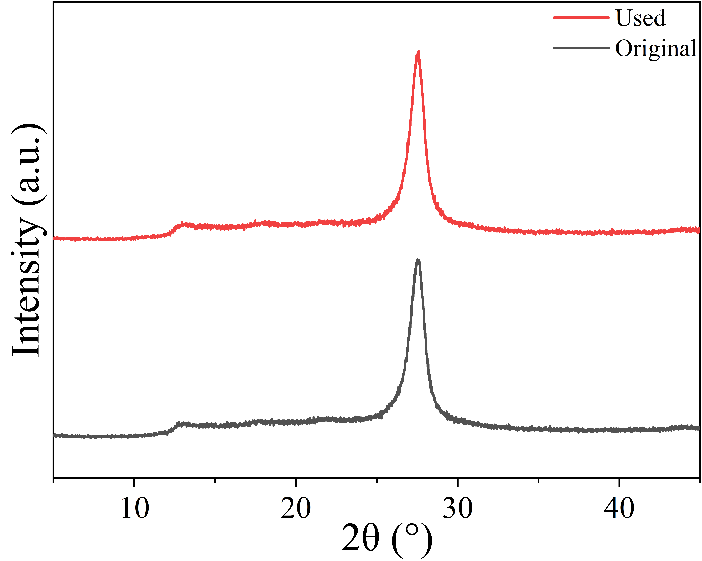


**Figure S11.** XRD patterns of CSC(50) before and after five consecutive photocatalytic runs.


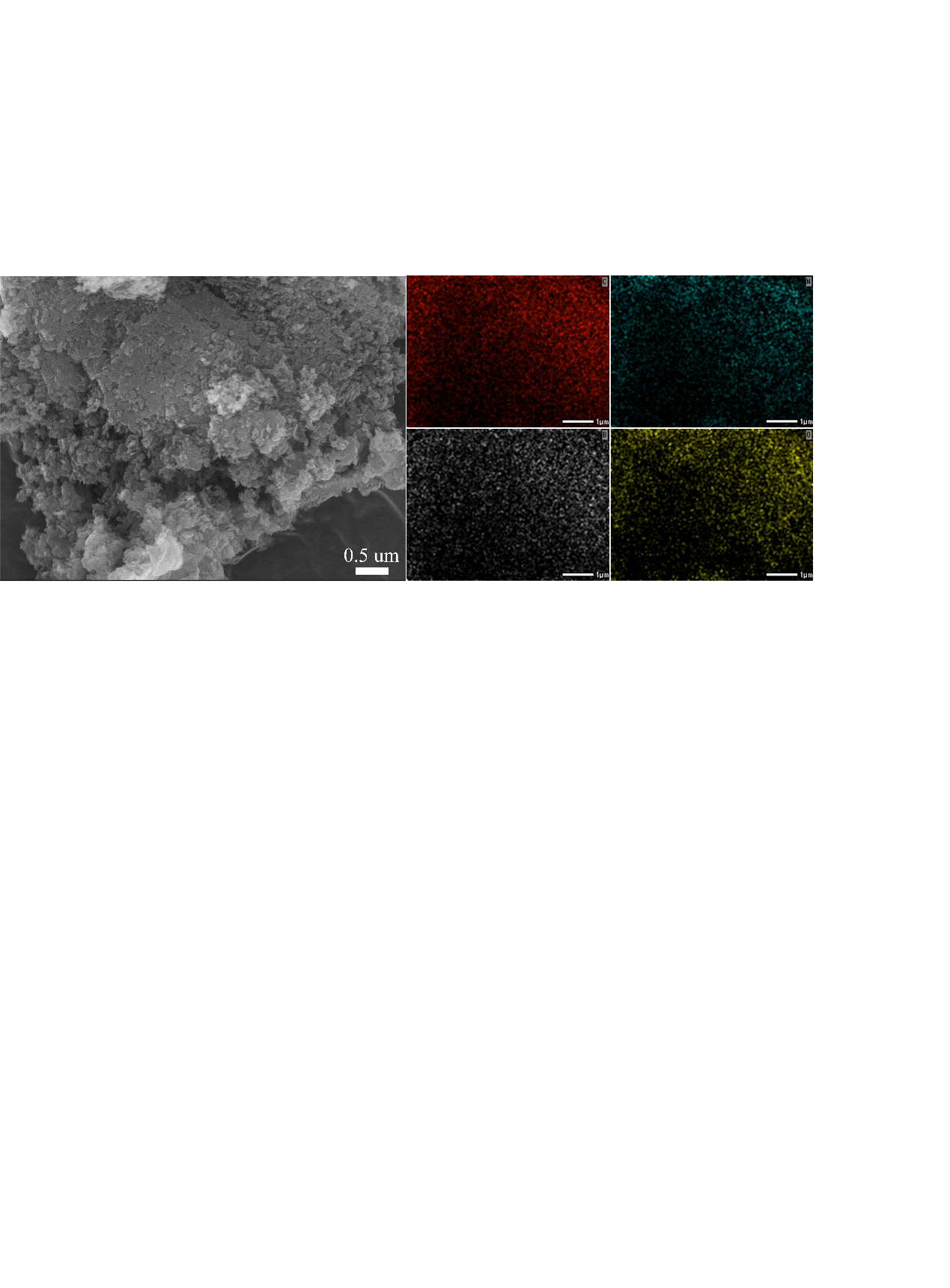


**Figure S12.** SEM and elemental mapping images of CSC(50) after five photocatalytic cycles.


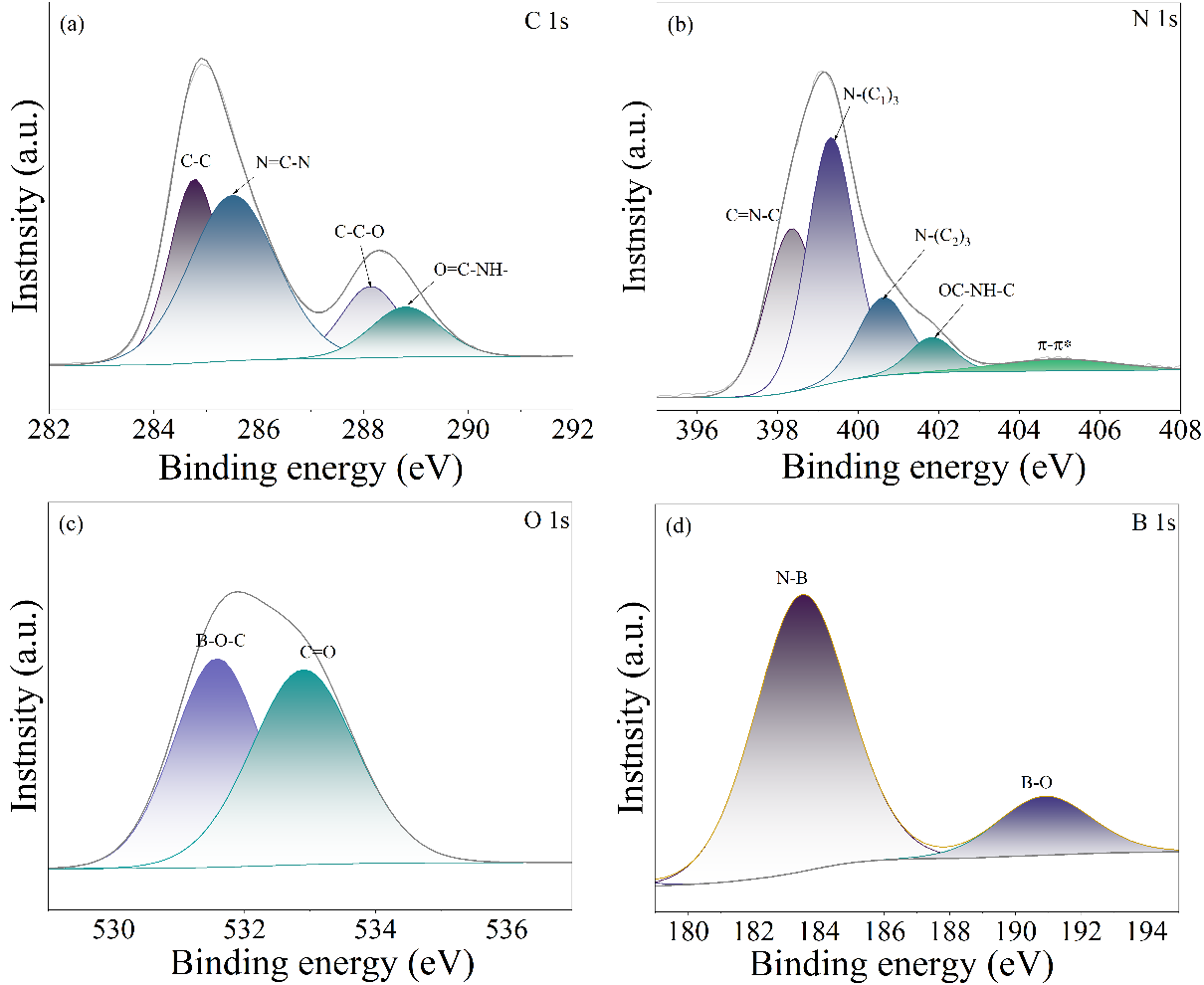


**Figure S13.** XPS spectrum of CSC(50) after 24-hour continuous photocatalytic reaction.


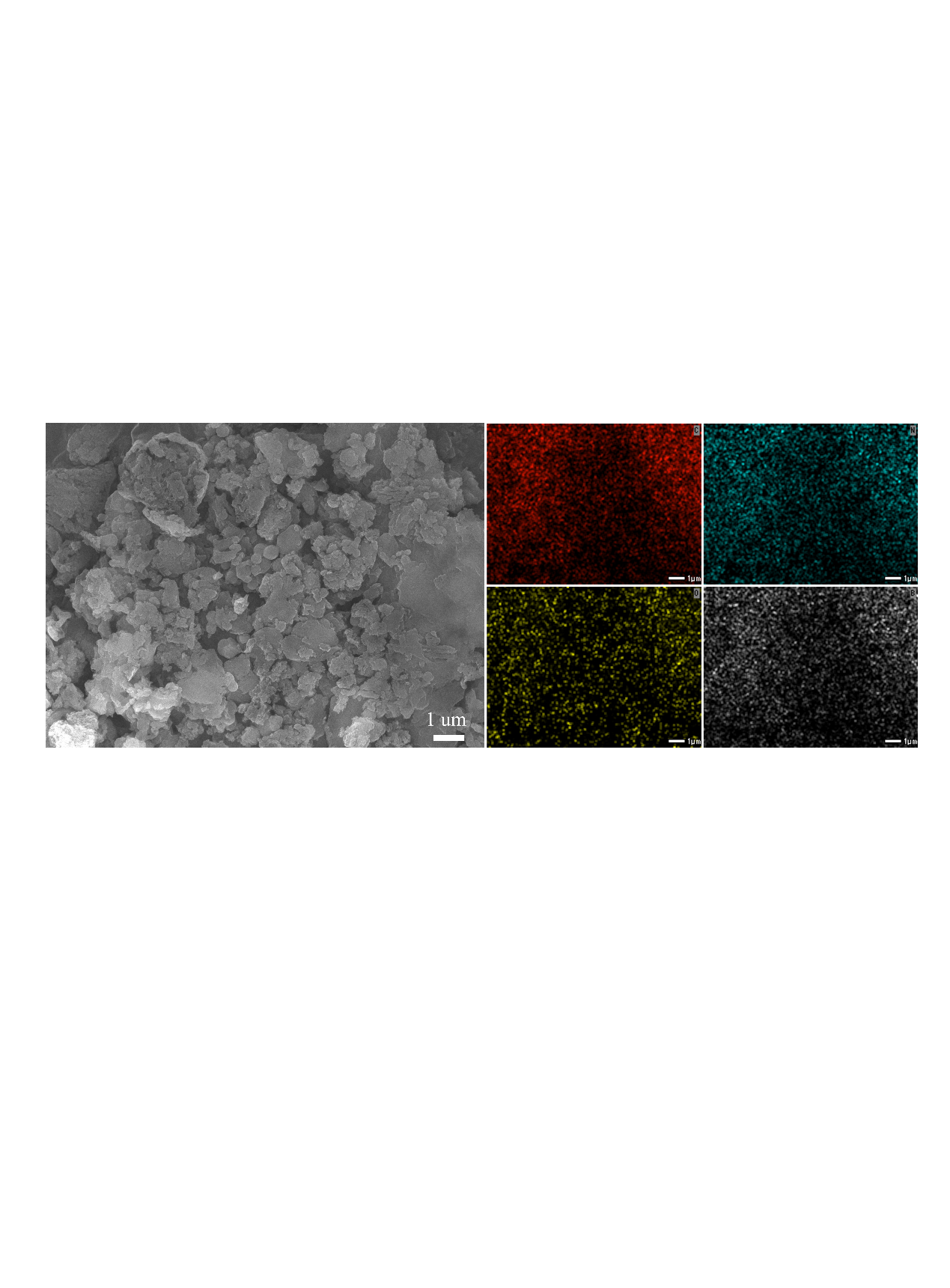


**Figure S14.** SEM and elemental mapping images of CSC(50) after 24-hour continuous photocatalytic reaction.

**
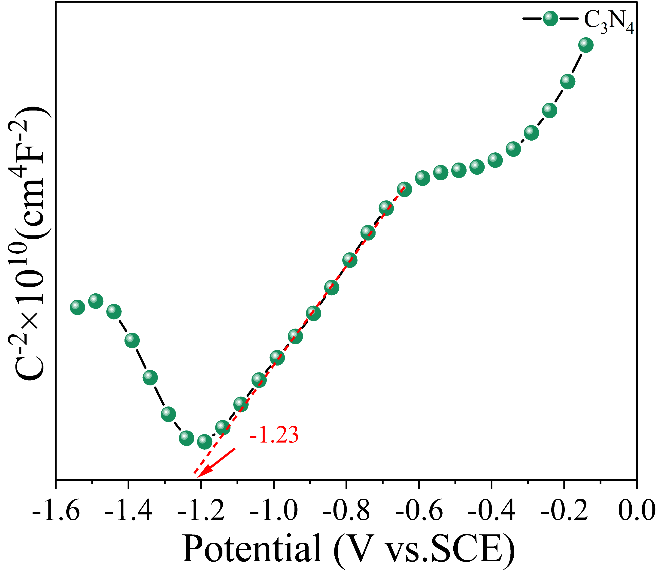
**

**Figure S15.** MottSchottky analysis of C_3_N_4_.


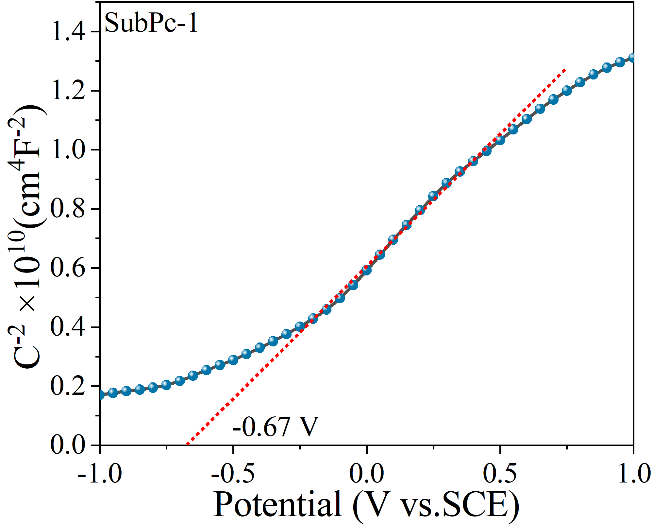


**Figure S16.** MottSchottky analysis of SubPc-1.


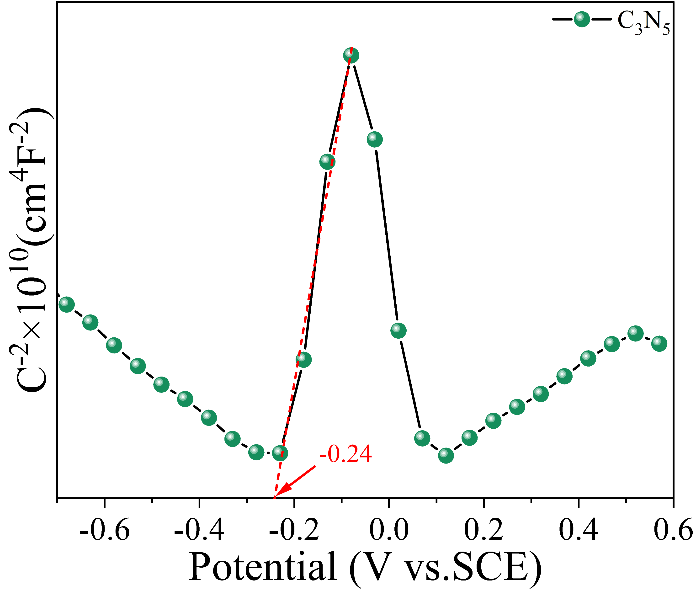


**Figure S17.** MottSchottky analysis of C_3_N_5_.


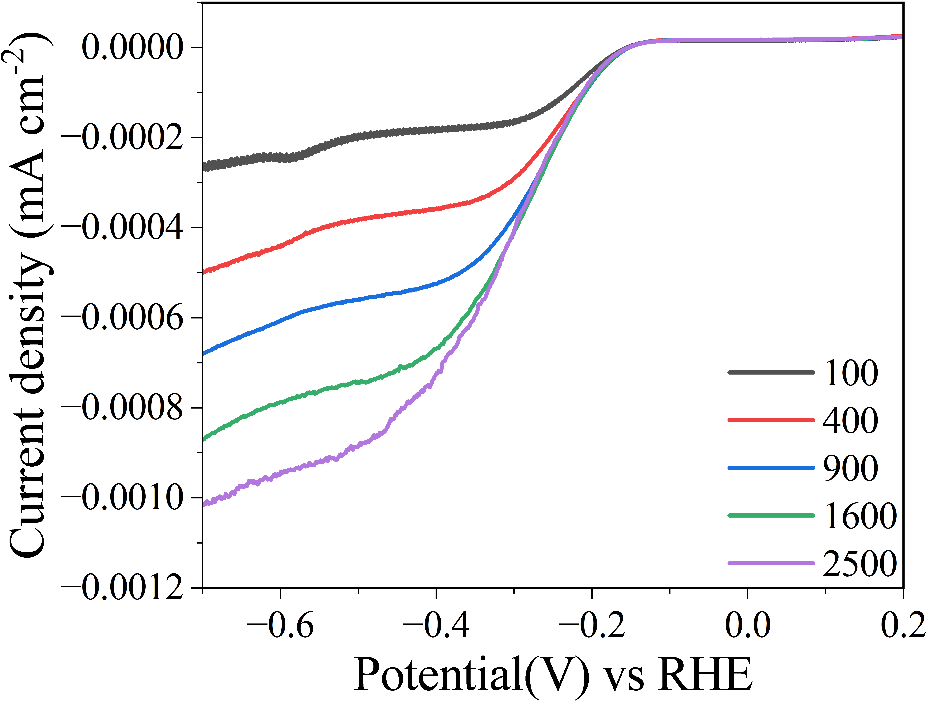


**Figure S18.** Linear sweep voltammetry (LSV) curves of the CSC(50) catalyst recorded in O₂-saturated 0.1 M KOH at different rotation rates (100 to 2500 rpm) with a scan rate of 10 mV s⁻¹.


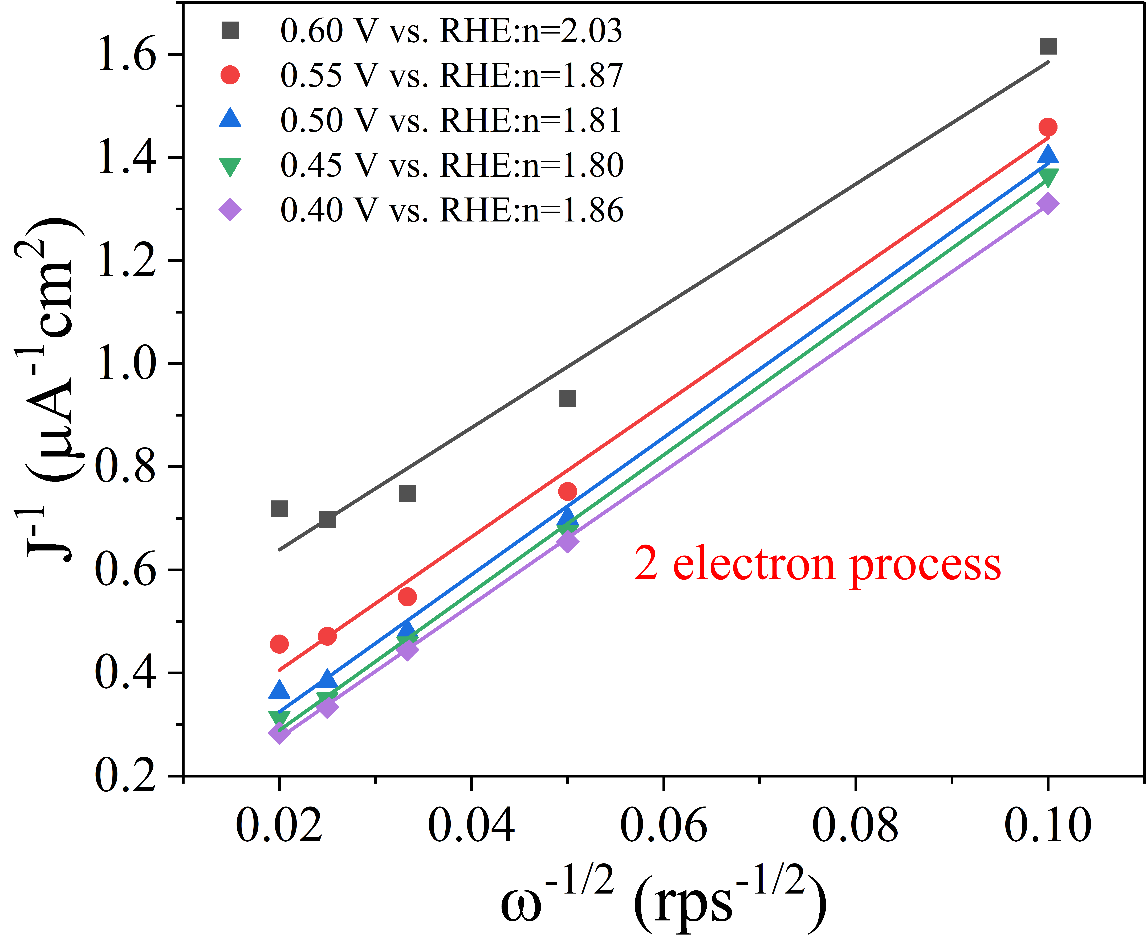


**Figure S19.** Koutecky-Levich plots derived from RDE measurements at different potentials (0.40 V, 0.45 V, 0.50 V, 0.55 V, 0.60 V vs. RHE) versus Ag/AgCl.

**
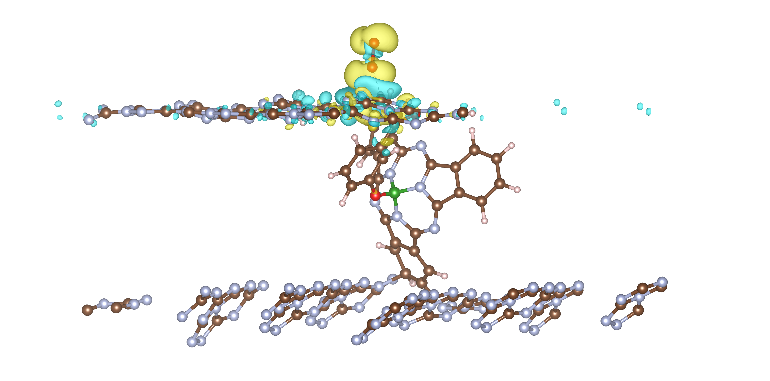
**

**Figure S20.** 3D charge-density difference of Pauling-type O_2_ adsorption model (isovalue ±0.001 e bohr⁻³): yellow, accumulation; cyan, depletion.

**
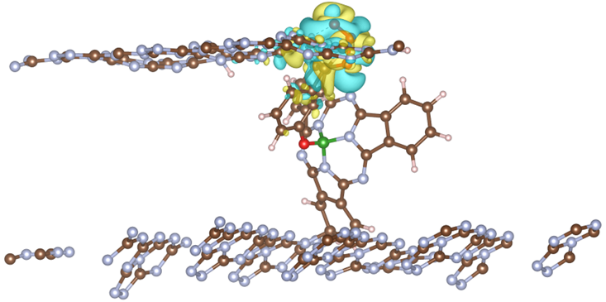
**

**Figure S21.** 3D charge-density difference of Yeager-type O_2_ adsorption model (isovalue ±0.001 e bohr⁻³): yellow, accumulation; cyan, depletion.

**
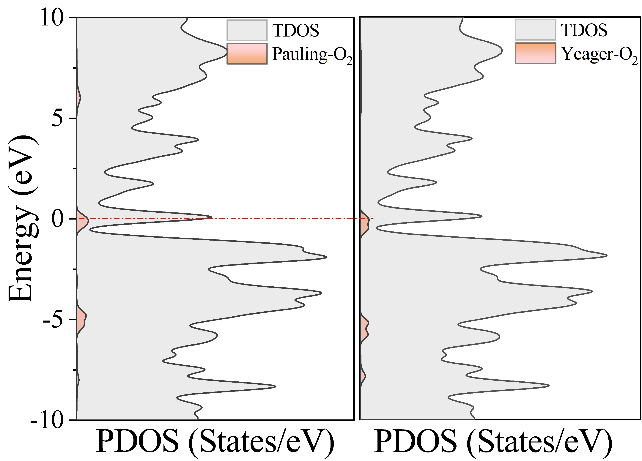
**

**Figure S22.** DOS/PDOS of Pauling-type and Yeager-type O_2_ adsorption model.

**
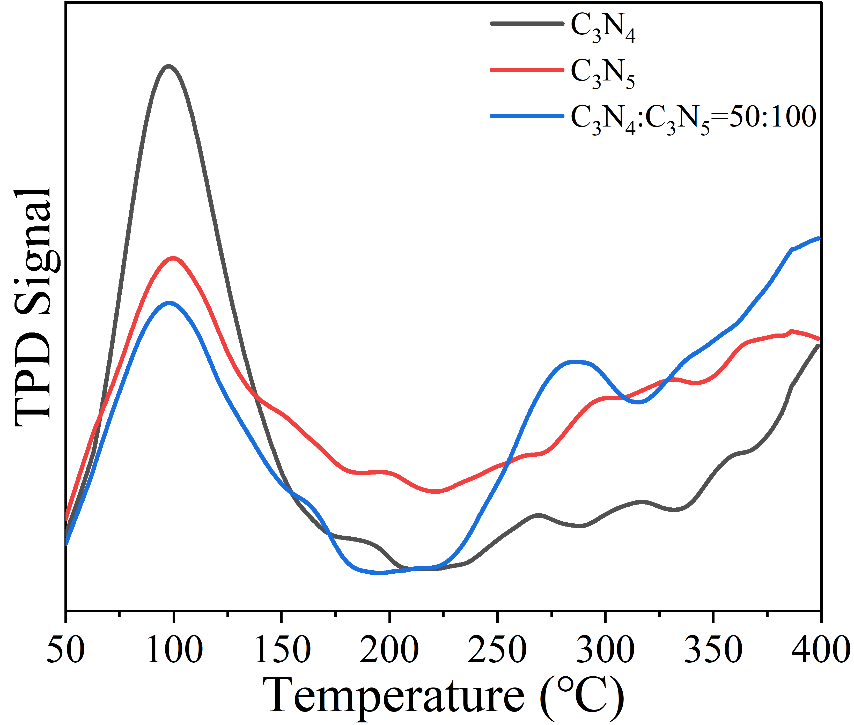
**

**Figure S23.** Oxygen temperature-programmed desorption (O₂-TPD) measurement of the as-prepared material.

**
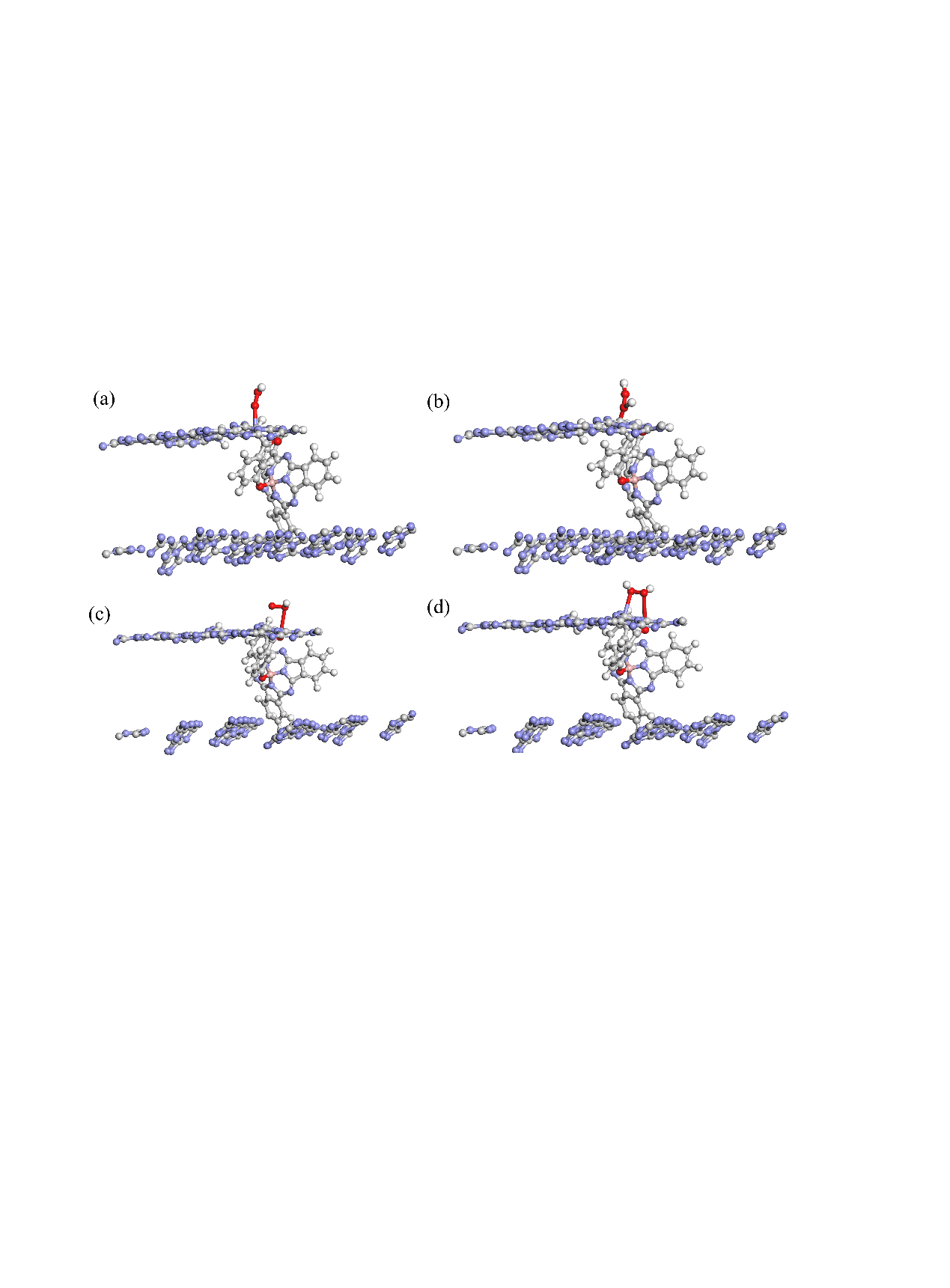
**

**Figure S24.** Optimized structural models:a) Pauling-type *OOH, b) Pauling-type *HOOH, c) Yeager-type *OOH, d) Yeager-type *HOOH.


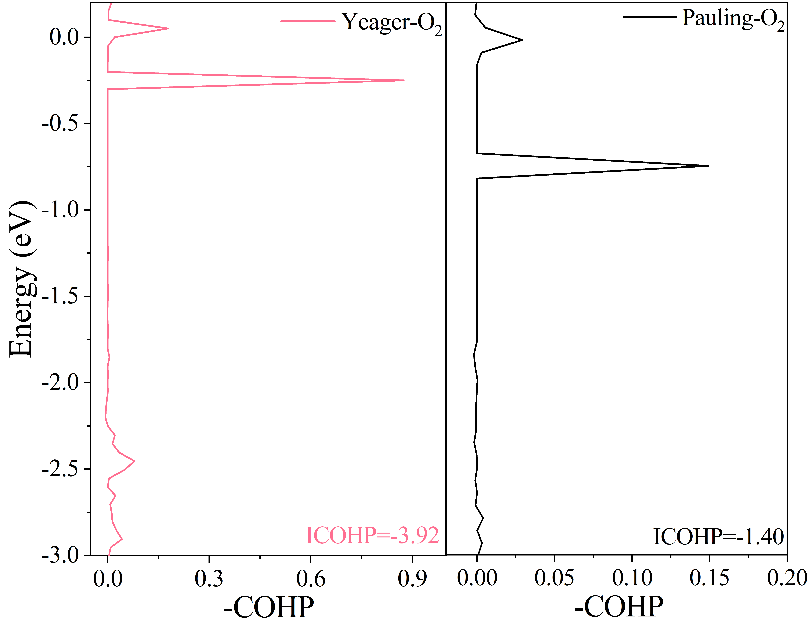


**Figure S25.** DFT-calculated ICOHP analyses of Yeager-type and Pauling-type O_2_ adsorption mode.

**
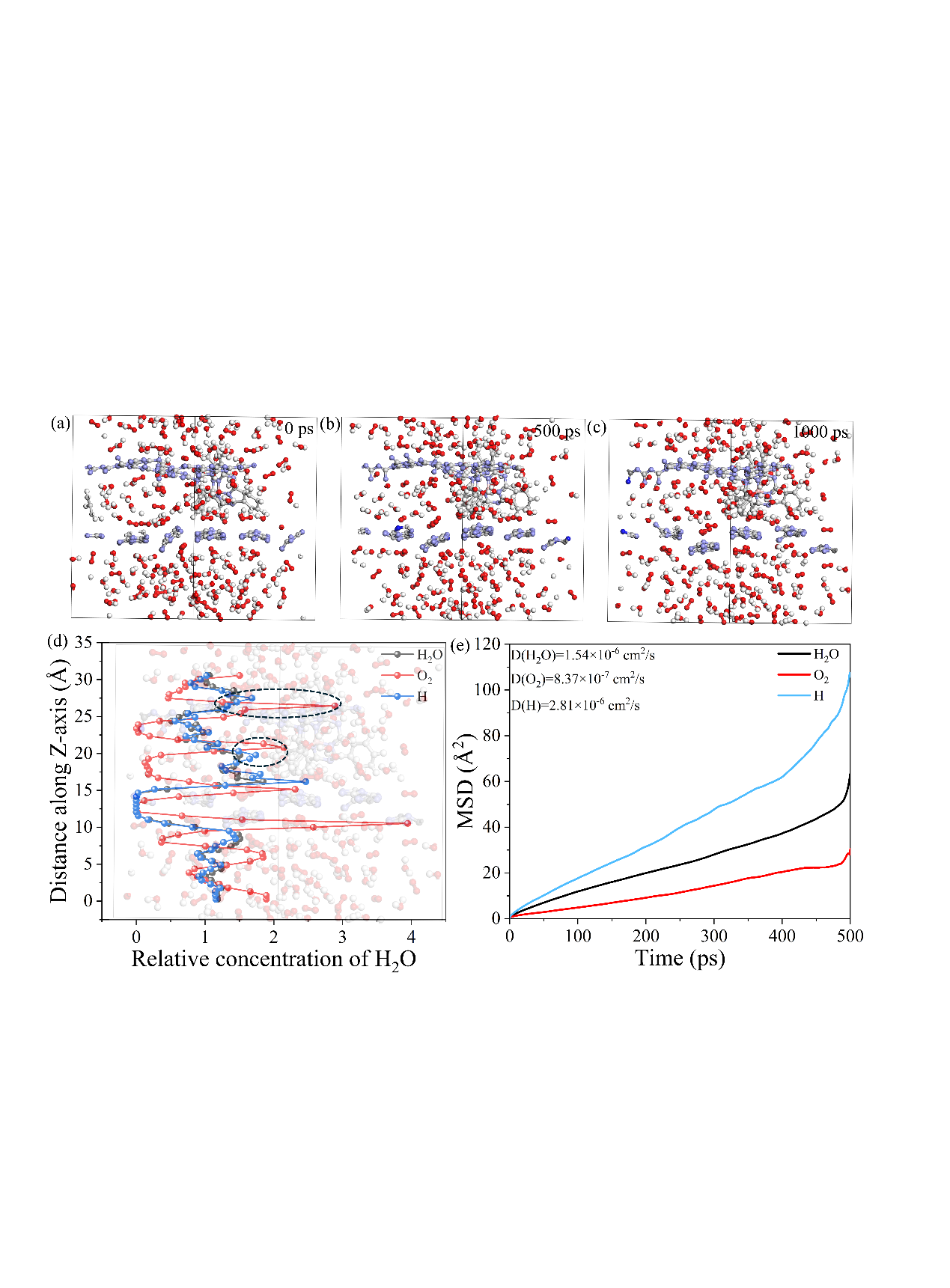
**

**Figure S26.** a–c) Snapshots of molecular-dynamics simulations for H₂O, O₂ and H on the CSC surface; d) Concentration profiles of H₂O, O₂ and H along the Z-direction; e) MSD curves and diffusion coefficients of H₂O, O₂ and H.

**
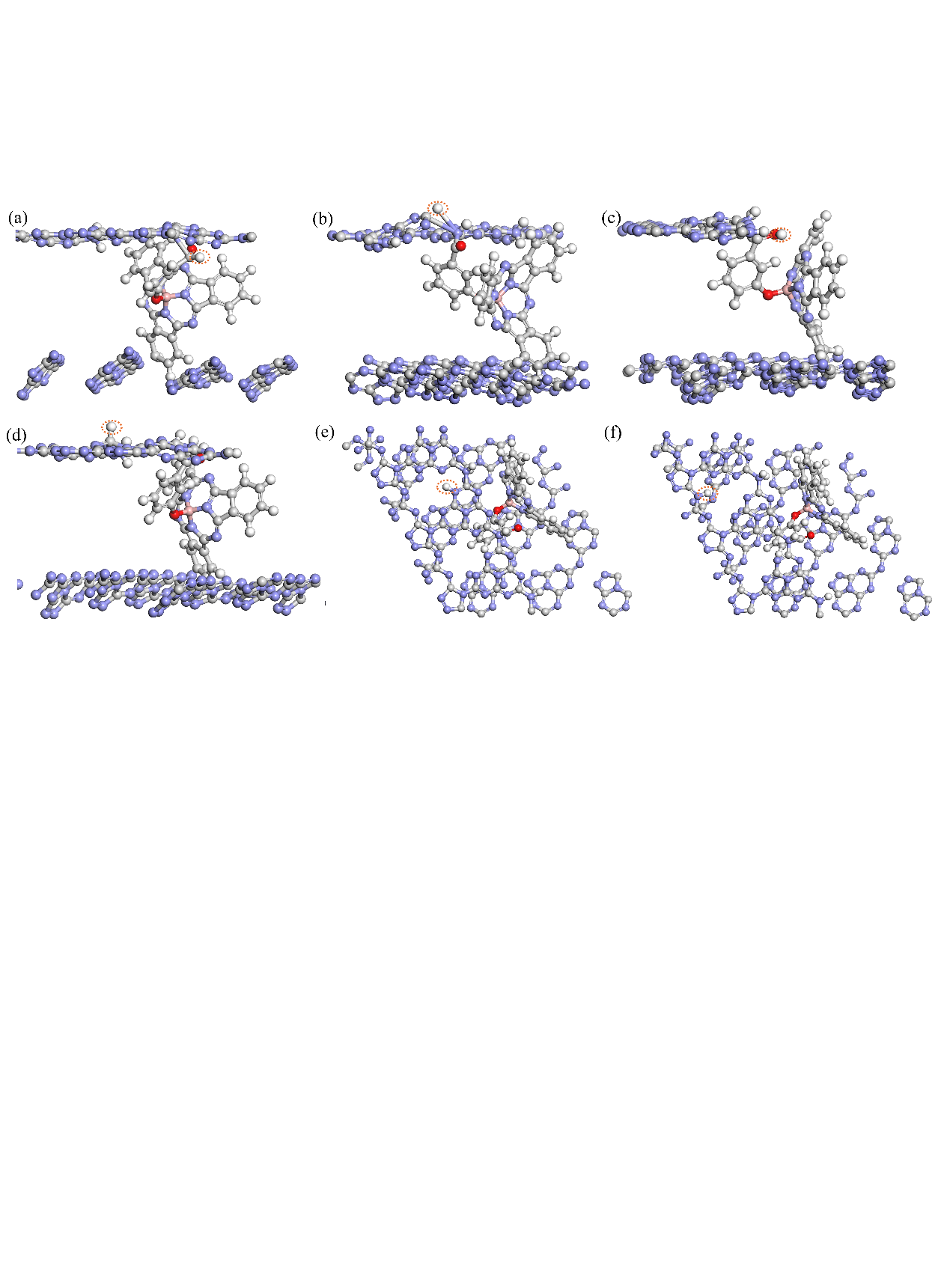
**

**Figure S27.** Optimized adsorption structures of *Hads on various sites of the CSC surface: a) C of the imine bond, b) N of the imine bond, c) O of the imine bond, d) C of C₃N₄, e) N-(C₁)₃ of C₃N₄, f) N-(C₂)₃ of C₃N₄.

**
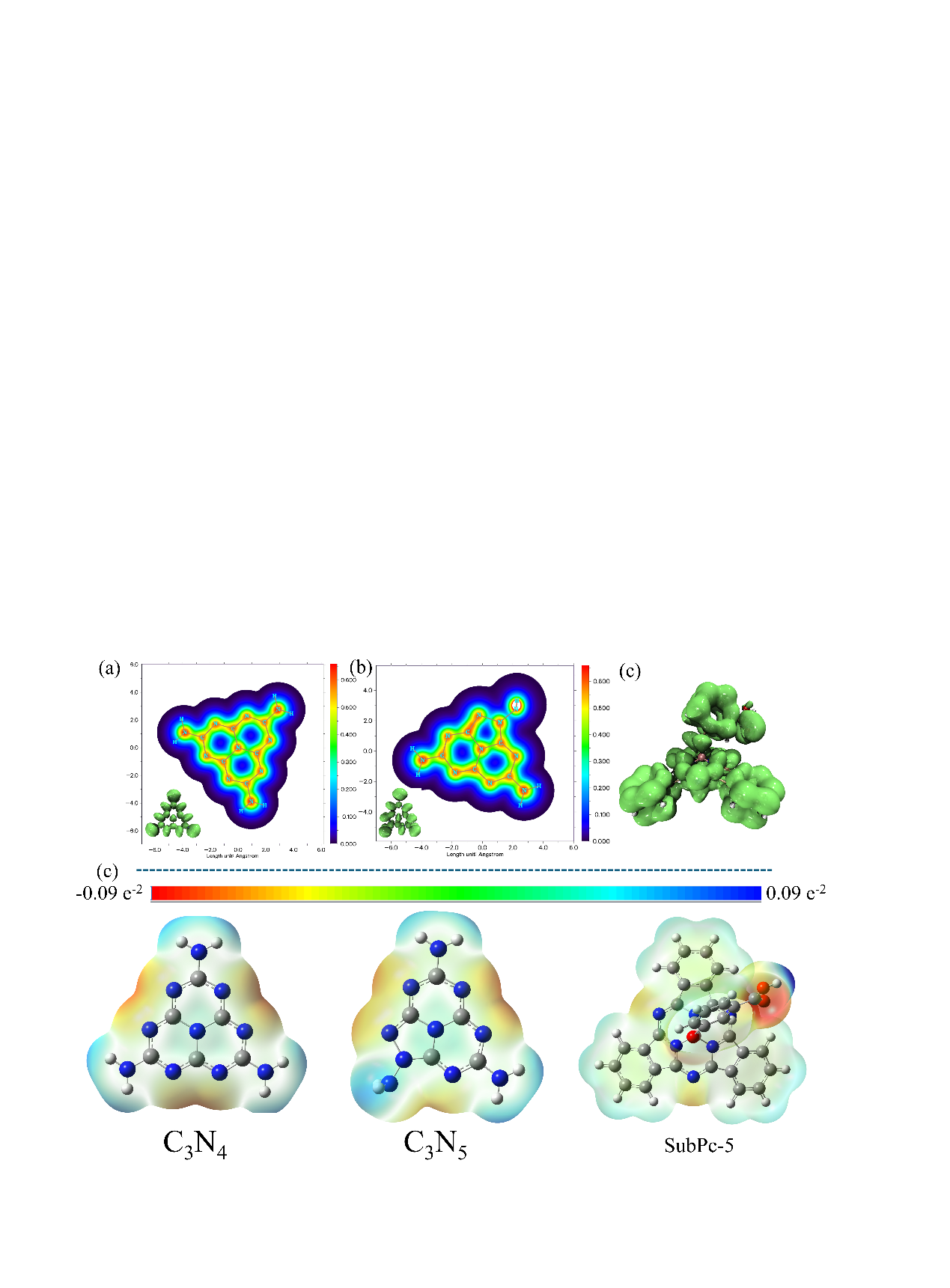
**

**Figure S28.** a) Localization of Orbital Locator–pi (LOL-pi) color-filled maps of C₃N₄ and b) C₃N₅ (insets show LOL-pi isosurfaces); c) LOL-pi isosurface of SubPc-5; d) electrostatic potential (ESP) isosurfaces of C₃N₄, C₃N₅ and SubPc-5.

**
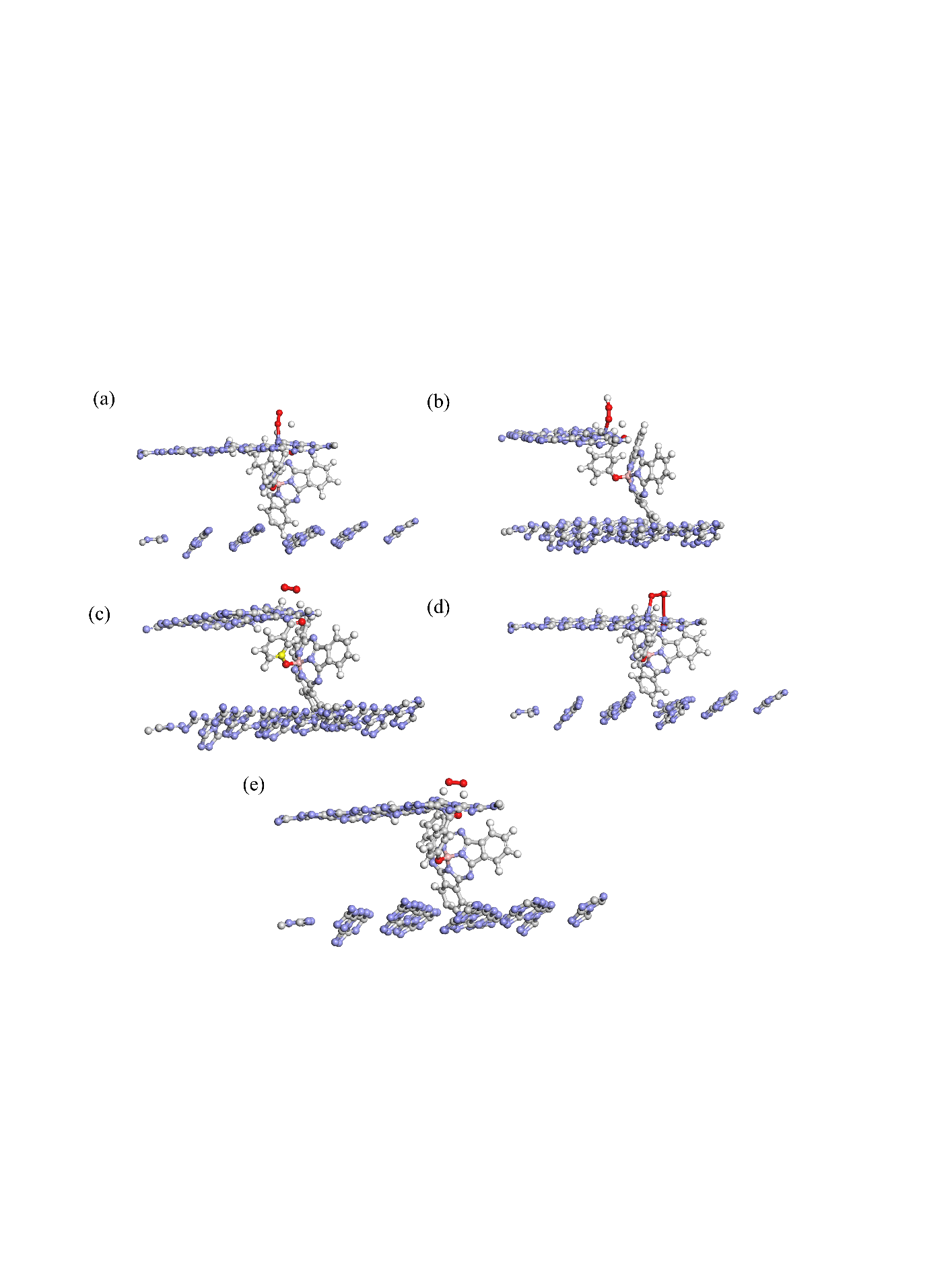
**

**Figure S29.** Optimized transition-state configurations: a) TS1 and b) TS2 of the Pauling-type O₂ adsorption model, d) TS1 and e) TS2 of the Yeager-type O₂ adsorption model, f) *O₂ → *HOOH on the Yeager-type O₂ adsorption model.

**
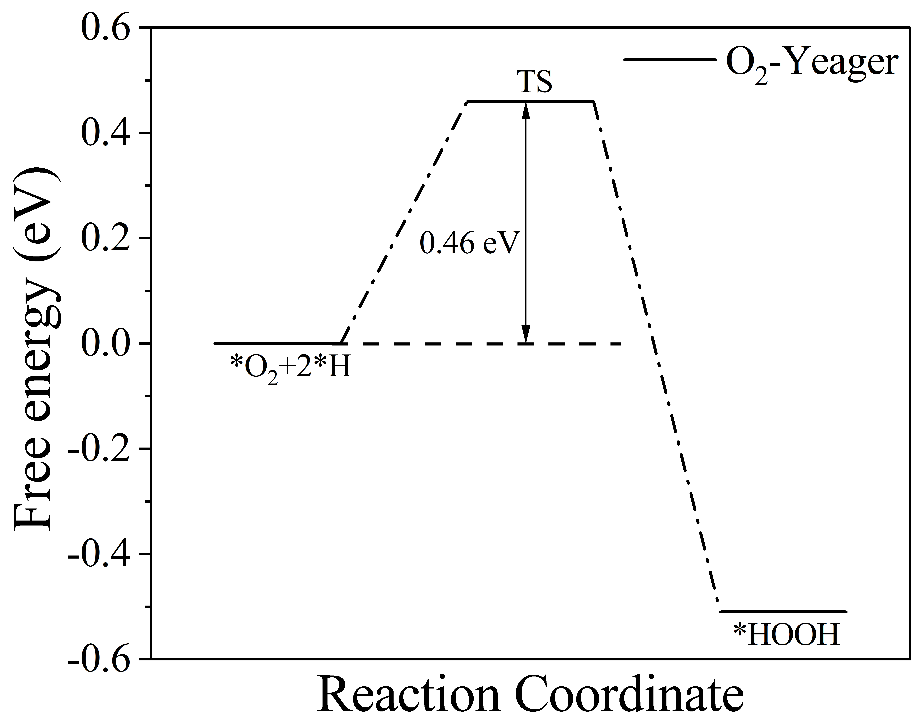
**

**Figure S30.** The energy barrier of *O_2_ to *HOOH on Yeager-type O_2_ adsorption model.


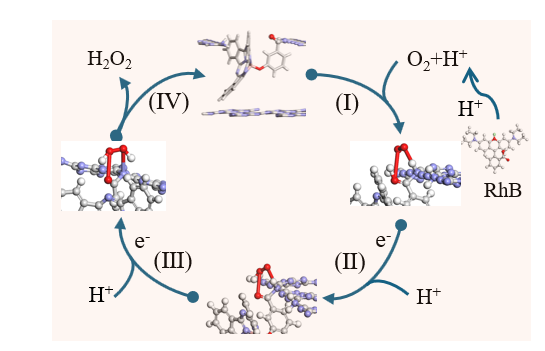


**Figure S31.** Catalytic cycle of the two-step 2e⁻ indirect ORR mechanism over the CSC(50) system.

**
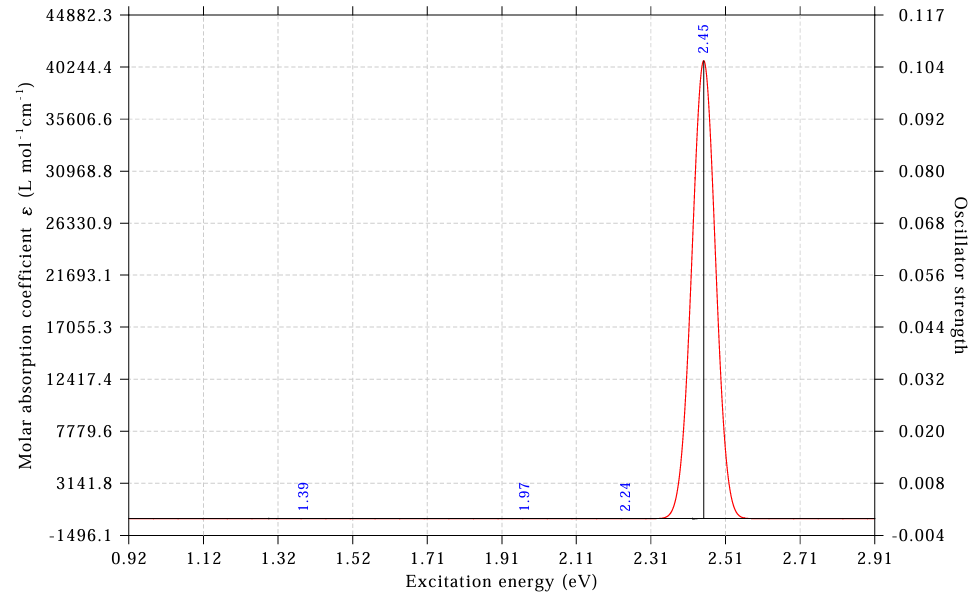
**

**Figure S32.** UV–vis spectrum of C_3_N_4_ obtained from TDDFT calculations.

**
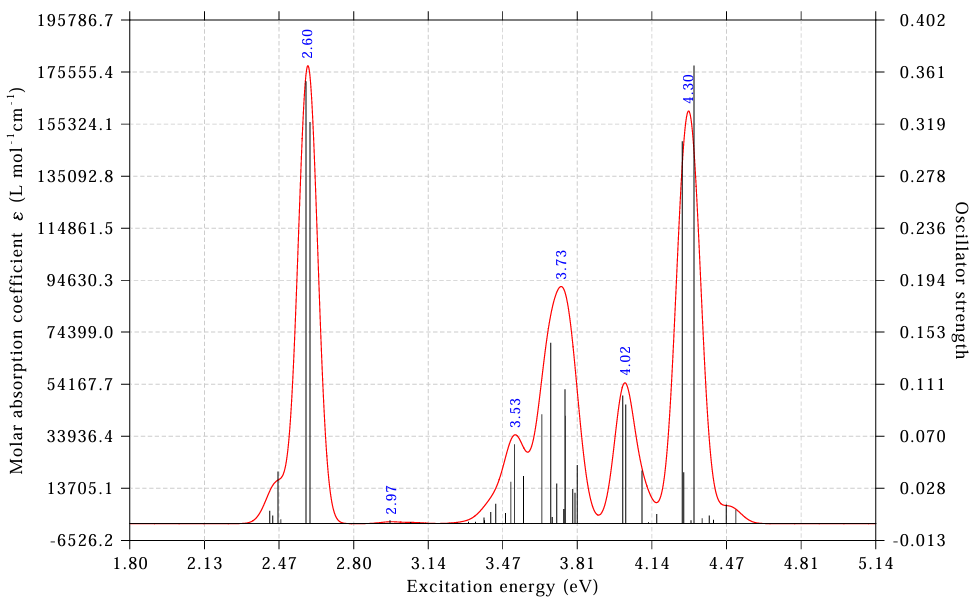
**

**Figure S33.** UV–vis spectrum of C_3_N_5_ obtained from TDDFT calculations.

**
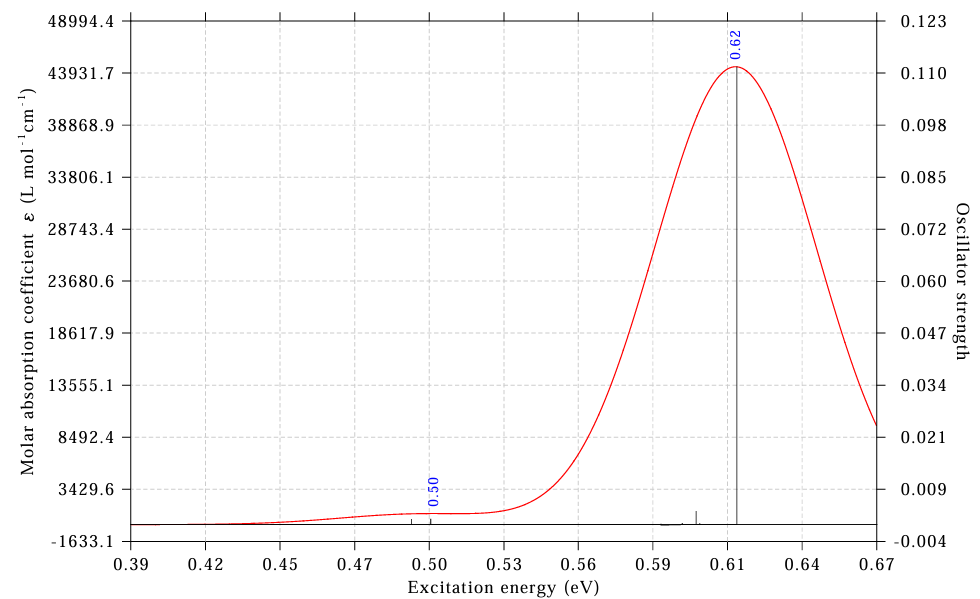
**

**Figure S34.** UV–vis spectrum of SubPc-1 obtained from TDDFT calculations.

**
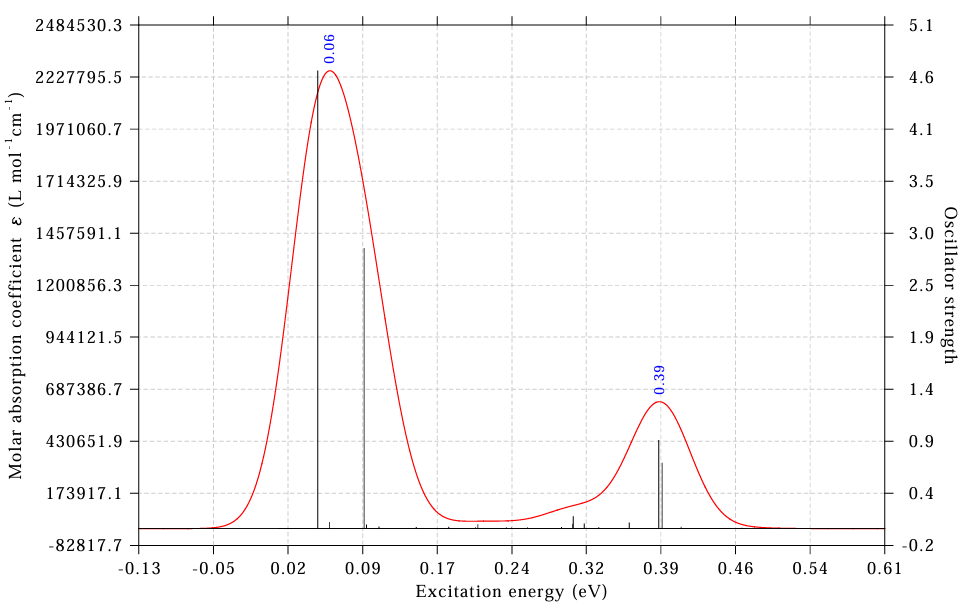
**

**Figure S35.** UV–vis spectrum of CSC composite obtained from TDDFT calculations.


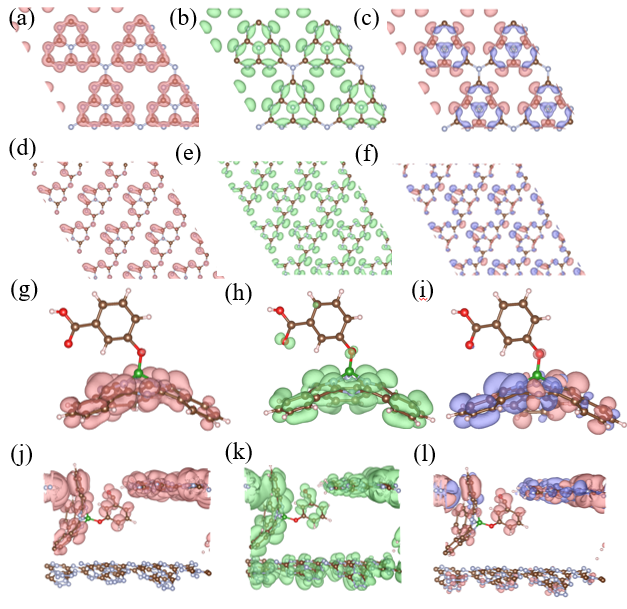


**Figure S36.** TDDFT-calculated isosurface plots (isovalue = 0.001 e bohr⁻³) for: a) electron, b) hole, c) transition density of C₃N₄; d) electron, e) hole, f) transition density of C₃N₅; g) electron, h) hole, i) transition density of SubPc-1; j) electron, k) hole, l) transition density of CSC.

**
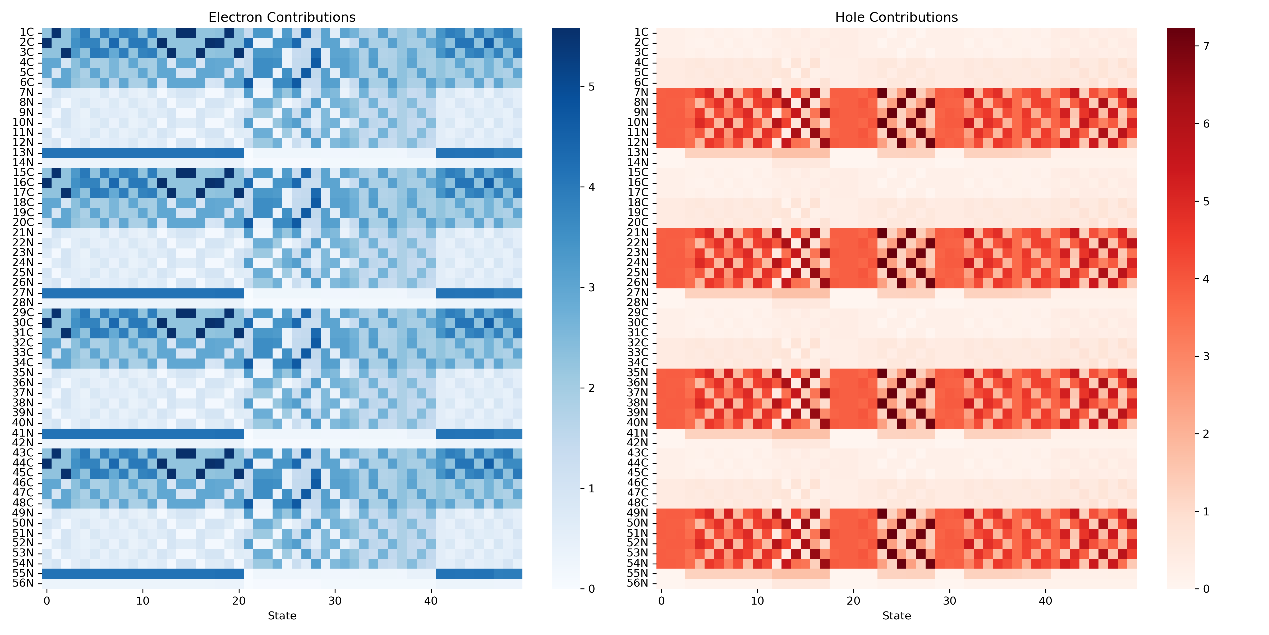
**

**Figure S37.** Heat map of atom-resolved electron and hole contributions for the lowest 50 excited states of C₃N₄ calculated by TDDFT.

**
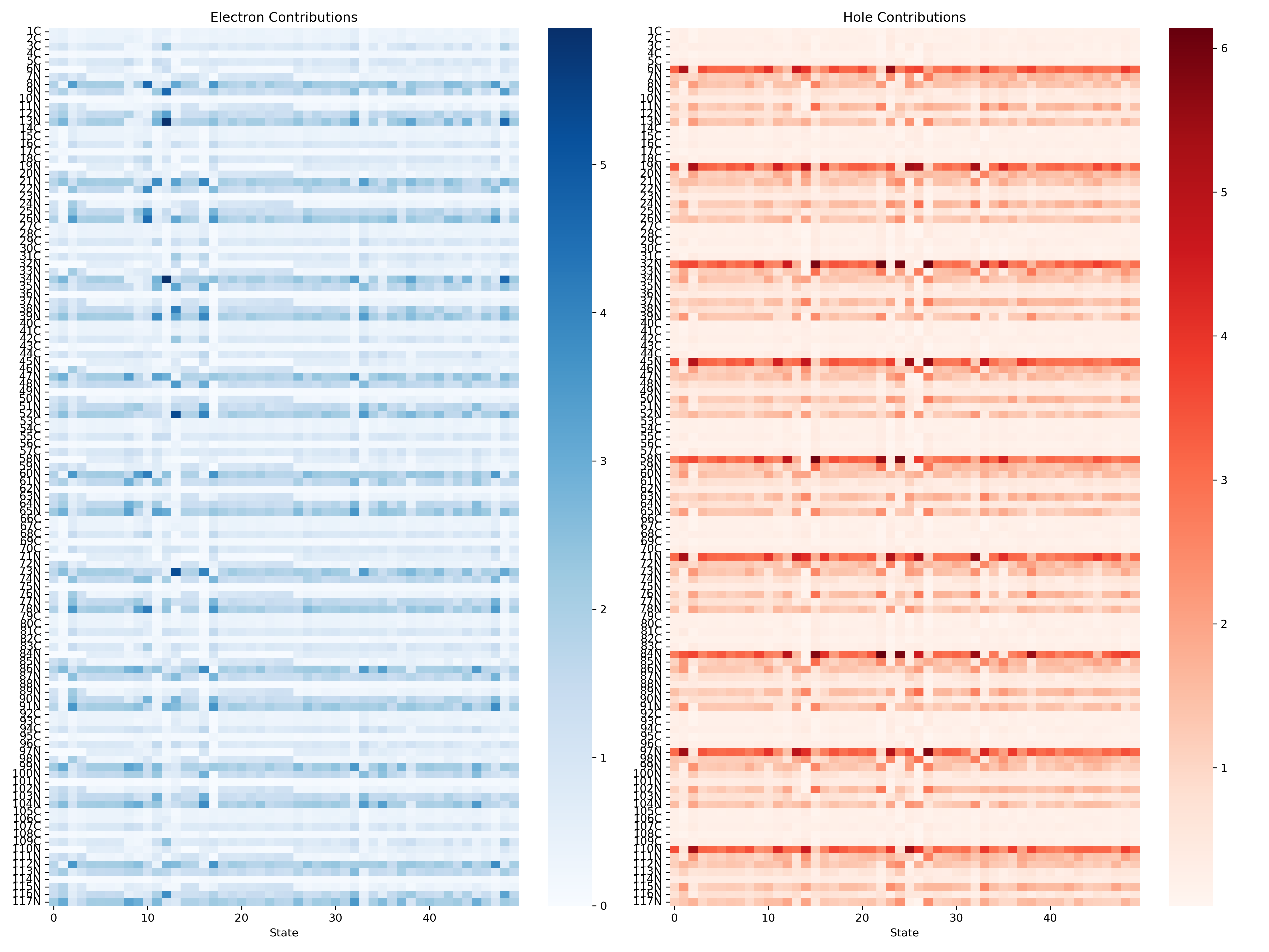
**

**Figure S38.** Heat map of atom-resolved electron and hole contributions for the lowest 50 excited states of C_3_N_5_ calculated by TDDFT.

**
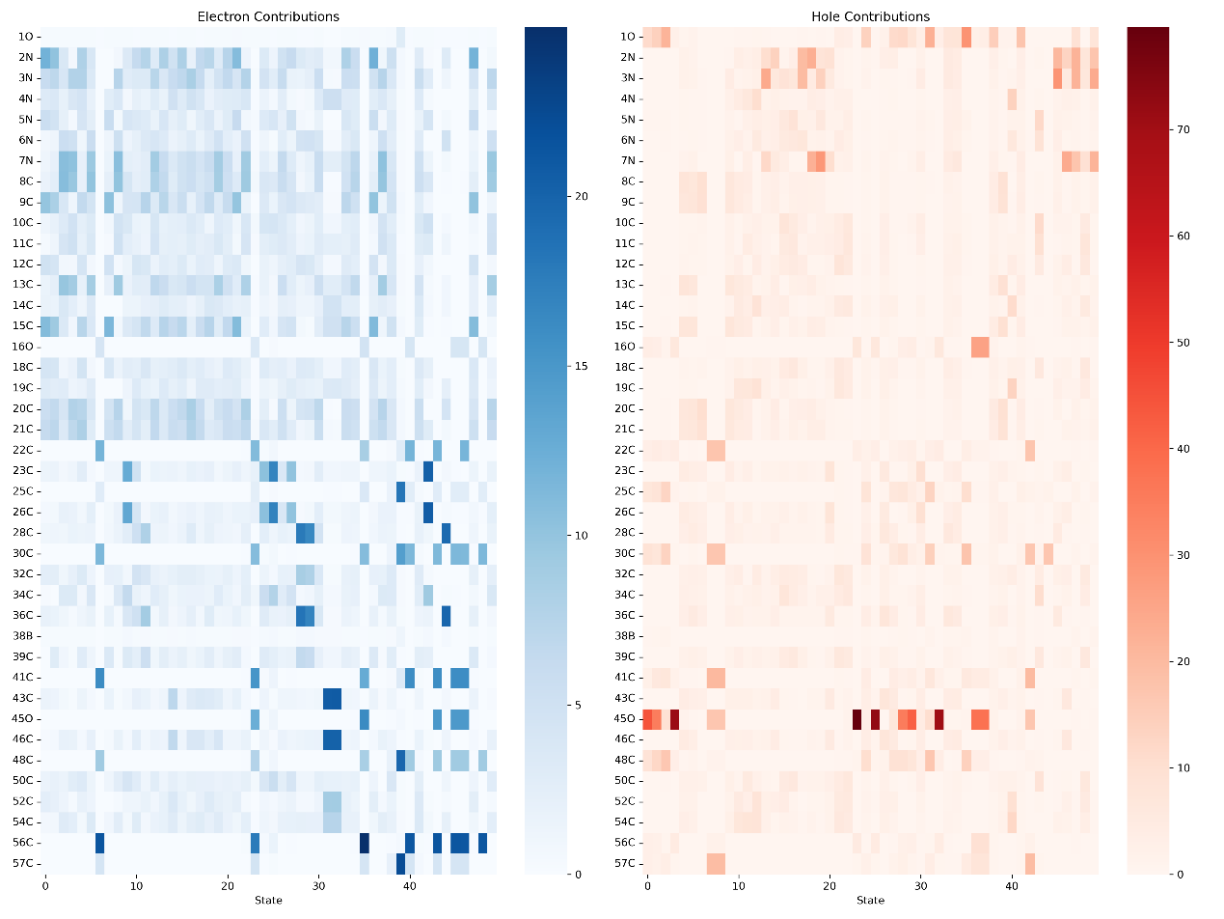
**

**Figure S39.** Heat map of atom-resolved electron and hole contributions for the lowest 50 excited states of SubPc-1 calculated by TDDFT.

**
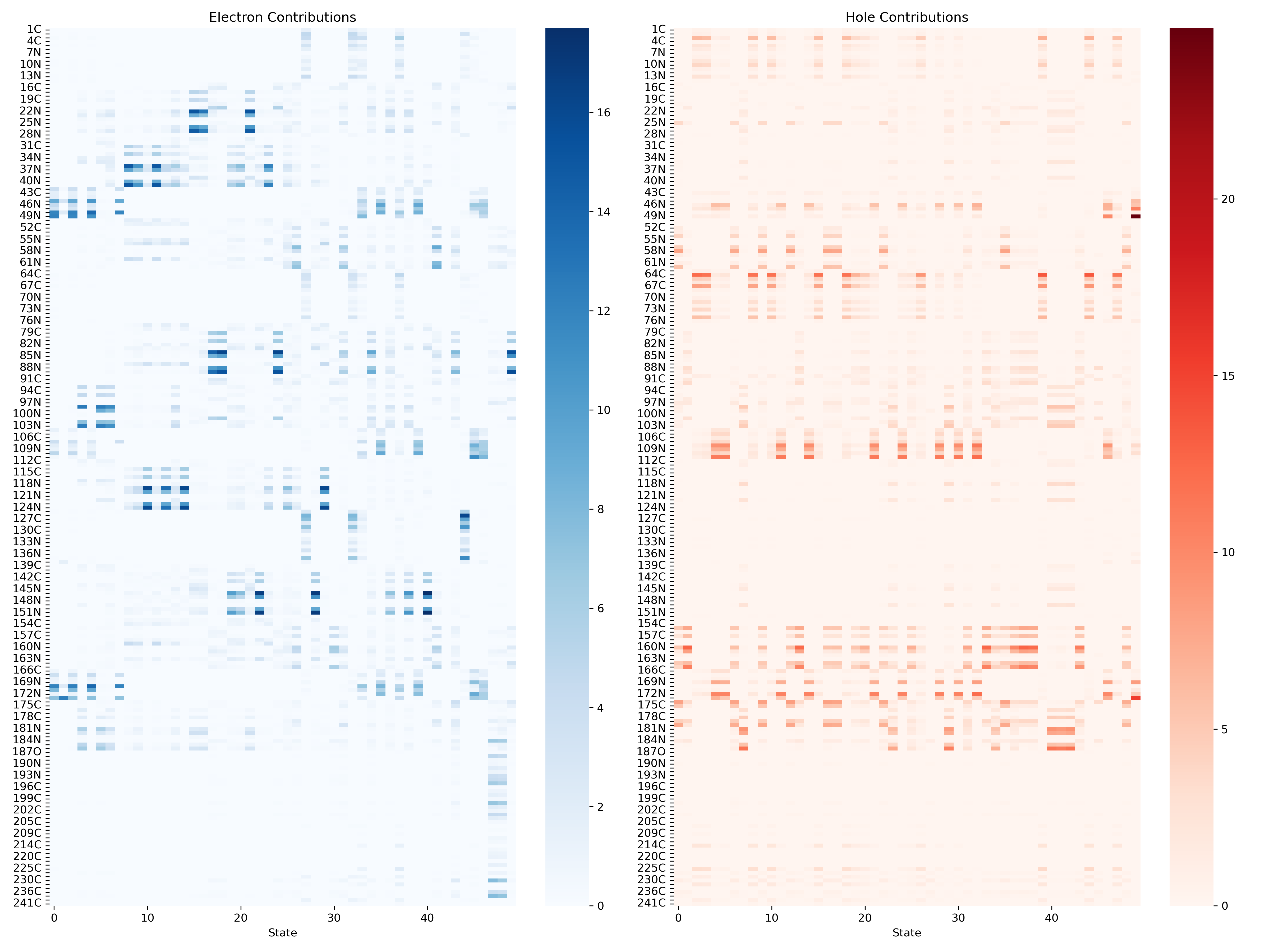
**

**Figure S40.** Heat map of atom-resolved electron and hole contributions for the lowest 50 excited states of CSC calculated by TDDFT.

**
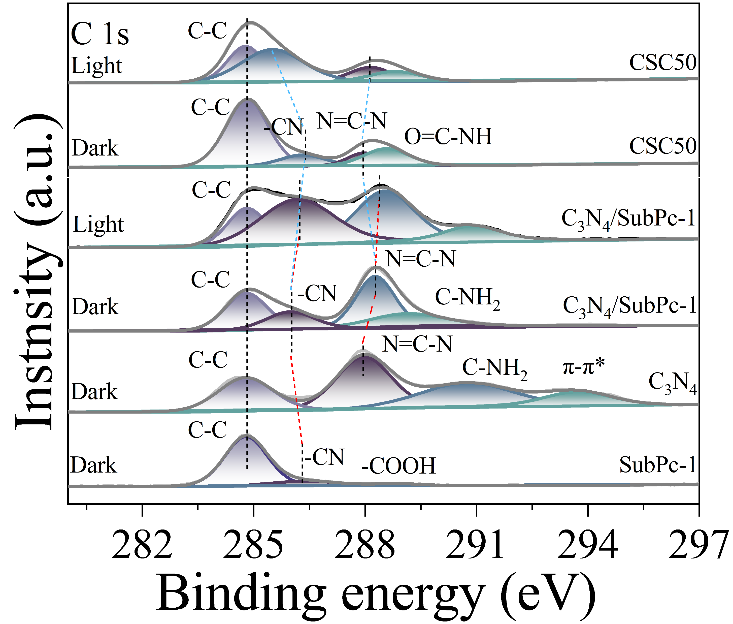
**

**Figure S41.** Comparative SI-XPS spectra of the C 1s region.

**
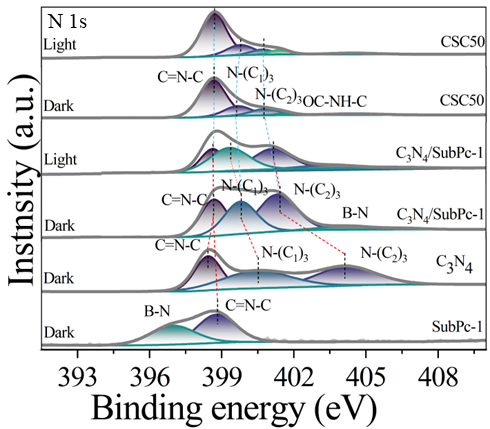
**

**Figure S42.** Comparative SI-XPS spectra of the N 1s region.

**
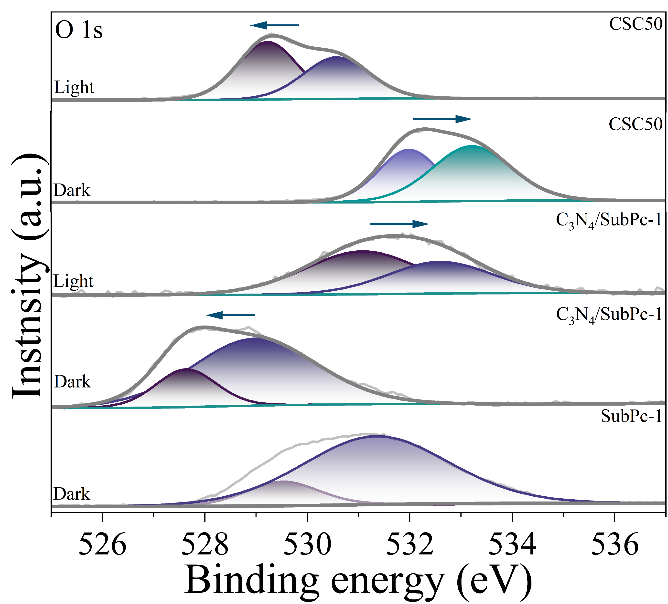
**

**Figure S43.** Comparative SI-XPS spectra of the O 1s region.

**References**

[1] Z. Li, B. Wang, B.-B. Zhang, H.-X. Ma, J. Xue, L. Xu, L.-Y. Jiao, X.-X. Ma, Journal of Photochemistry and Photobiology A: Chemistry, **2021,** *405*, 112929.

[2] P. Chao, B. Wang, W. Luo, E. Liu, H. Wu, L. Jiao, J. Li, H. Ma, C. Dai, Z. Li, Applied Catalysis B: Environment and Energy, **2025,** 125694.

[3] X. Zhang, H. Su, P. Cui, Y. Cao, Z. Teng, Q. Zhang, Y. Wang, Y. Feng, R. Feng, J. Hou, Nat. Commun., **2023,** *14*, 7115.

[4] B. Civalleri, C.M. Zicovich-Wilson, L. Valenzano, P. Ugliengo, CrystEngComm, **2008,** *10*, 405-410.

[5] J. Hafner, J. Comput. Chem., **2008,** *29*, 2044-2078.

[6] V. Wang, N. Xu, J.-C. Liu, G. Tang, W.-T. Geng, Comput. Phys. Commun., **2021,** *267*, 108033.

[7] H.-F. Wang, Z.-P. Liu, J. Am. Chem. Soc., **2008,** *130*, 10996-11004.

[8] C. Shang, Z.-P. Liu, J. Chem. Theory Comput., **2010,** *6*, 1136-1144.

[9] X.-J. Zhang, C. Shang, Z.-P. Liu, J. Chem. Theory Comput., **2013,** *9*, 5745-5753.

[10] J. Hutter, M. Iannuzzi, F. Schiffmann, J. VandeVondele, Wiley Interdisciplinary Reviews: Computational Molecular Science, **2014,** *4*, 15-25.

[11] T. Lu, F. Chen, J. Comput. Chem., **2012,** *33*, 580-592.
